# Structure and mechanism of the two-component α-helical pore-forming toxin YaxAB

**DOI:** 10.1038/s41467-018-04139-2

**Published:** 2018-05-04

**Authors:** Bastian Bräuning, Eva Bertosin, Florian Praetorius, Christian Ihling, Alexandra Schatt, Agnes Adler, Klaus Richter, Andrea Sinz, Hendrik Dietz, Michael Groll

**Affiliations:** 1 Center for Integrated Protein Science Munich (CIPSM), Department of Chemistry, Chair of Biochemistry, Technische Universität München, Lichtenbergstrasse 4, 85747 Garching, Germany; 2 Center for Integrated Protein Science Munich (CIPSM), Department of Physics, Technische Universität München, Am Coulombwall 4a, 85748 Garching, Germany; 3 Department of Pharmaceutical Chemistry and Bioanalytics, Institute of Pharmacy, Martin-Luther University Halle-Wittenberg, Wolfgang-Langenbeck-Str.4, 06120 Halle/Saale, Germany; 4 Center for Integrated Protein Science Munich (CIPSM), Department of Chemistry, Chair of Biotechnology, Technische Universität München, Lichtenbergstrasse 4, 85747 Garching, Germany

## Abstract

Pore-forming toxins (PFT) are virulence factors that transform from soluble to membrane-bound states. The Yersinia YaxAB system represents a family of binary α-PFTs with orthologues in human, insect, and plant pathogens, with unknown structures. YaxAB was shown to be cytotoxic and likely involved in pathogenesis, though the molecular basis for its two-component lytic mechanism remains elusive. Here, we present crystal structures of YaxA and YaxB, together with a cryo-electron microscopy map of the YaxAB complex. Our structures reveal a pore predominantly composed of decamers of YaxA–YaxB heterodimers. Both subunits bear membrane-active moieties, but only YaxA is capable of binding to membranes by itself. YaxB can subsequently be recruited to membrane-associated YaxA and induced to present its lytic transmembrane helices. Pore formation can progress by further oligomerization of YaxA–YaxB dimers. Our results allow for a comparison between pore assemblies belonging to the wider ClyA-like family of α-PFTs, highlighting diverse pore architectures.

## Abstract (web summary)

The Yersinia YaxAB system is a pore-forming toxin of so far unknown structure. Here authors present X-ray and cryo-EM to structures of individual subunits and of the YaxAB pore complex, and find that YaxA binds to membranes first and recruits YaxB for subsequent oligomerization.

## Introduction

Pore-forming toxins (PFTs) are found ubiquitously throughout prokaryotic and eukaryotic domains of life, contributing centrally to virulence and defense^1, 2^. Decades of research on PFT structure and function have produced rich mechanistic understanding for numerous families of these proteins^1^. Depending on the secondary structure of the membrane-perforating pore, PFTs can be partitioned into α-PFTs and β-PFTs. PFTs bury their membrane-active domains in the soluble state and expose them upon encountering a receptor molecule on the host cell surface^3–8^. In most PFTs, these hydrophobic moieties become part of the membrane pore structure, in the form of an oligomeric α-helical or β-barrel type channel^1^.

The majority of PFTs characterized so far via X-ray crystallography^8–12^ and cryogenic single-particle electron microscopy (cryo-EM)^13–15^ are homooligomeric pore assemblies. In contrast, there are fewer structural studies on PFTs producing heterooligomeric pores. One example is the heterooctameric staphylococcal γ-haemolysin, the structure of which features an α-haemolysin-like β-barrel transmembrane pore^16^. The two protein components share some 30% sequence identity and undergo similar conformational transitions from soluble to pore states. Another example is pleurotolysin, a two-component β-PFT from the edible oyster mushroom^17, 18^. Here, the membrane-binding and pore-forming components (PlyA and PlyB, respectively) are structurally unrelated, as shown in a recent study^7^. Prominent examples of heterooligomeric α-PFTs are the large tripartite insecticidal toxin complexes (Tc)^19–21^. These syringe-like assemblies consist of a homopentameric pore-forming subunit (TcA), and the TcB/TcC pair, which docks onto the TcA pore and contains the ADP-ribosyltransferase toxin cargo to be injected into susceptible cells^21^. Other heteromeric α-PFTs include Hbl and Nhe, tripartite cytotoxins suggested to contribute to foodborne illnesses caused by *Bacillus cereus*^22–24^. Based on sequence and structural similarities to the well-characterized α-PFT Cytolysin A (ClyA)^12^, the toxin components Hbl-B and NheA from *B. cereus* were classified within the ClyA family of α-PFTs^25, 26^.

The binary XaxAB toxin—discovered in entomopathogenic *Xenorhabdus nematophila* as a PFT^27^—represents a structurally uncharacterized family of α-PFT, with XaxA bearing low sequence similarity to ClyA and Hbl-B. Nonetheless, sequential action of XaxA and XaxB on susceptible membranes in vitro holds true for all orthologues analyzed to date^27–29^, similar to the Hbl and Nhe toxin systems^30^. XaxAB represents one of the main cytolytic factors released into *X. nematophila* culture media, strongly cytotoxic to insect hemocytes^27^. Expressed toward later stages of the bacteria’s life cycle within the insect host, XaxAB is thought to be required for the breakdown of the larval cadaver^31^. Recently, the possible involvement of a XaxAB orthologue during course of infection within a mammalian host was reported^28^. Moreover, a strain of the human pathogen *Y. enterocolitica*, in which the toxin orthologue YaxAB was knocked out, showed altered colonization behavior and tissue pathology in a murine infection model. Potentially a virulence factor, YaxAB was found to be strongly upregulated by the Yersinia master regulator RovA^32^, but further characterization, especially regarding the structure of this PFT, is still missing. Here, we present crystal structures of YaxA (45.9 kDa) and YaxB (39.3 kDa) from *Y. enterocolitica*, as well as PaxB (40.6 kDa) from *Photorhabdus luminescens*. The pore complex, which we studied by cryo-EM, displays a predominant decamer of dimers (20-mer) arrangement of its two components. Conserved protein regions involved in membrane binding and cytolysis were confirmed through structure-guided mutagenesis, clarifying the necessity for a bipartite assembly and allow us to propose a plausible pathway of pore assembly. Finally, our structures enable the first direct comparison between pores of the wider ClyA family of α-PFTs, highlighting diverse toxin architectures.

## Results

### YaxA and YaxB orthologues exhibit distinct helical folds

**Fig. 1:**
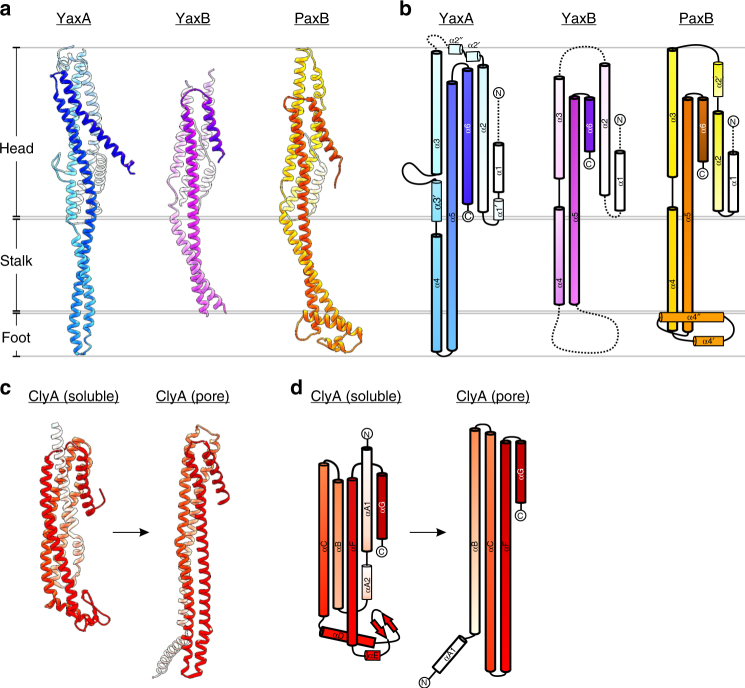
Structures of YaxA, YaxB, and the YaxB orthologue PaxB. **a** Crystal structures of YaxA (blue), YaxB (pink), and the YaxB orthologue PaxB (orange). Ribbons are shaded from light (N-terminus) to dark (C-terminus). All three proteins partition into a helix bundle head domain, a pronounced coiled-coil stalk and a foot domain. Two helices α4′ and α4″ of PaxB comprise the lytic moiety of the toxin and will be mentioned throughout the manuscript. **b** Topology diagrams of YaxA, YaxB, and PaxB colored according to **a**. α1-α6 in bold black outline denote the structural frame common to both toxin subunits YaxA and YaxB/PaxB. Helices in thinner black outline are unique for the respective protein.  Dotted lines signify protein regions unresolved in the crystal structures. **c** Crystal structures of ClyA in its soluble form (PDB code 1QOY) and as a pore-protomer (PDB code 2WCD). The cartoons are to-scale with those in **a**. **d** Topology diagrams of ClyA in soluble and pore-protomeric states colored according to **c**. Helices are assigned according to previous nomenclature^12^. Helices boldly outlined in black correspond putatively to helices α1-α6 of the YaxAB system illustrated in **b**

We expressed recombinant YaxA and YaxB, as well as PaxB from *P. luminescens*, in *Escherichia coli* and purified the proteins to homogeneity for structural and biochemical purposes. The orthologue PaxB produced better diffracting crystals than YaxB and thus served as template for molecular replacement phasing (the two proteins share 40% amino acid sequence identity, whereas YaxA shares 18% and 22% sequence identity with PaxB and YaxB, respectively). YaxA and PaxB crystal structures were determined using single-wavelength anomalous dispersion (SAD) on selenomethionine-derivatized proteins (Fig. 1a). The resolutions achieved were 1.8 Å for YaxA (*R*_free_ = 22.8%, PDB code 6EK7) and 2.8 Å for PaxB (*R*_free_ = 26.8%, PDB code 6EK4). Subsequently, we obtained a medium-resolution crystal structure of YaxB at 4 Å (*R*_free_ = 33.9%, PDB code 6EK8). Despite high *B*-factors in some protein regions, especially for YaxA, electron density maps were clearly interpretable for all three crystal structures (Supplementary Figure 1). Our structural data confirmed that YaxB is a true homolog to PaxB, with a RMSD of 2.1 Å across 210 C_α_ pairs (Fig. 1a and Supplementary Figure 2a). Because our PaxB model could be well resolved, missing merely the N-terminal 11 residues, we will utilize these coordinates for most structural analyses throughout the manuscript. All three proteins adopt similarly elongated, α-helical folds (Fig. 1a) composed of a five-helix bundle head domain and a two-helix-coiled-coil stalk, tapering to a narrow turn in the case of YaxA, while connecting to an additional α-helical domain in the case of PaxB. We refer to these latter regions as foot domains, which are the most striking features of discrimination between YaxA and PaxB. Notably, the YaxB foot domain could not be resolved in the 2Fo–Fc electron density map and therefore remains unmodeled. These foot domains from both toxin subunits comprise the membrane-active moieties of the toxin and will be referred to throughout the manuscript.

As seen in Supplementary Figure 2b, the head domains of YaxA and PaxB superpose well (RMSD of 2.9 Å across 159 C_α_ pairs); topologically, both orthologues share a structural frame of helices α1-α6 (Fig. 1b, helices with bold black outline). We found striking similarities between both head domains, with all structurally elucidated members from the wider ClyA family of α-PFTs, by performing DALI^33^ analysis (Supplementary Figure 3a and Supplementary Figure 4a). For alignments of the YaxA head domain, *Z*-scores ranged from 9.6 (soluble-monomeric ClyA, 5.5 Å RMSD; PDB code 1QOY^34^) to 12.2 (NheA, 3.4 Å RMSD; PDB code 4K1P^26^). For the PaxB head domain, *Z*-scores ranged from 7.4 (soluble-monomeric ClyA, 3.8 Å RMSD) to 13.3 (pore-protomeric ClyA, 2.4 Å RMSD; PDB code 2WCD^12^). A comparison of topologies between the ClyA structures and YaxA/PaxB (Fig. 1c, d) reveals the following putative secondary structural correspondences: αA1-α1, αB-α2, αC-α3, αD-α4, αF-α5, and αG-α6 (ClyA helix nomenclature adapted from previous work^12^). However, despite clear homology between the YaxA/PaxB head domains with ClyA-like PFTs, the extended coiled-coil and foot domains distinguish the former proteins (Supplementary Figure 3b and Supplementary Figure 4b). In summary, YaxA and YaxB orthologues possess a ClyA-like head domain, while the coiled-coil and foot domains differ. Therefore, we propose that this class of toxins defines a new structural family of α-PFTs.

### YaxA and YaxB form a large complex in vitro

**Fig. 2:**
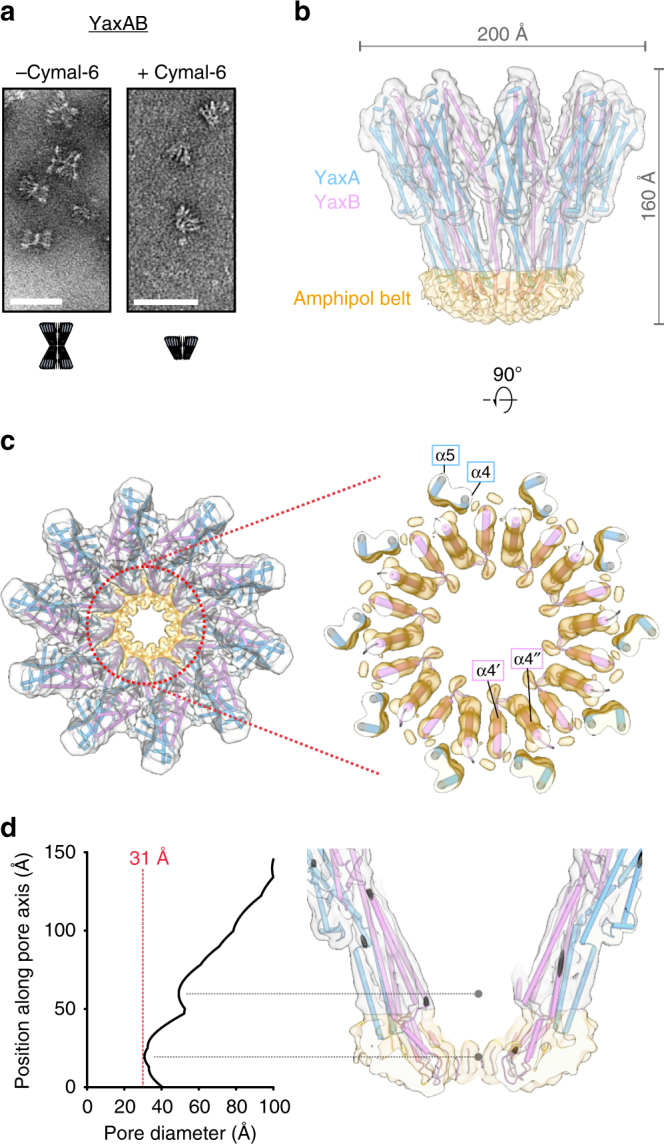
Cryo-EM structure of the YaxAB complex reveals an α-helical transmembrane pore. **a** Negative-stain TEM micrographs of YaxAB complexes in solution. Left: sample in absence of detergent. Right: sample in presence of 0.1% Cymal-6. Scale bar = 50 nm. **b** Final sharpened cryo-EM density of YaxAB together with the fitted pore model, shown from side view. The chosen contour level (4.5σ) corresponds to the 1.21 Å^3^/Dalton convention according to Harpaz et al.^65^. Fitted YaxA and YaxB models are colored blue and pink, respectively. The amphipol belt, illustrated in gold, is clearly visible and demarcates the putative transmembrane region. **c** Top view of the pore complex, overlaid with the final cryo-EM map. YaxA and YaxB form outer and inner rings, respectively (left). A zoom of the amphipol-enclosed portion of the complex (right) is contoured at 6.5σ to better distinguish individual helices. Labels of the secondary structure elements adhere to the nomenclature shown in Fig. 1b. **d** Pore diameter plotted against the coordinate along the vertical axis. Calculations have been performed using the program HOLE^66^ (left). The red line indicates the narrowest point in the channel. Two major constrictions along the YaxAB model are emphasized (right)

Direct interaction between YaxA and YaxB orthologues from *X. nematophila* was demonstrated using pull-down assays^27^. Upon mixing purified YaxA and YaxB, a high-molecular weight complex forms in solution, as we saw in size-exclusion chromatography experiments (SEC; Supplementary Figure 5a). The resulting YaxAB assembly exhibit a 1:1 ratio between YaxA and YaxB, as evaluated by SDS-PAGE band intensities. In negative-stain TEM micrographs, the YaxAB complex appeared like a pair of base-stacked crowns (Fig. 2a). Treatment of YaxAB with the mild detergent Cymal-6 resulted in complexes that no longer aggregated, suggesting that dimerization is caused through membrane-active regions in the absence of detergent (Fig. 2a and Supplementary Figure 5b, c). We found that YaxA’s foot domain was responsible for this detergent-dependent aggregation, as the absence of this region produced non-aggregating YaxAB particles (Supplementary Figure 5d). Following initial characterization, we proceeded with further TEM analyses to resolve the architecture of the complex in more detail.

### Architecture of the YaxAB complex

We considered two routes to obtain reconstituted pores for further structural studies by cryo-EM. Initially, we assembled the YaxAB pore complex on human erythrocyte membranes, followed by detergent extraction (Supplementary Figure 6). Negative-stain TEM images revealed heterogeneity in the stoichiometry of the YaxAB complex. The number of radial spokes observed in top-view particles ranged from 8 to 12, with 10 spokes being seen most frequently (Supplementary Figure 6e). In our negative-stain 3D reconstruction, we see apparent C11 symmetry (Supplementary Figure 6f). This mismatch between most abundant top-view and reconstruction symmetries may be due to preferred orientations of the particles on the EM grid. Despite its low resolution, this map clearly distinguishes an upper, spoked rim, from which density converges at a lower, cup-like funnel.

In our second approach, the complex was treated in solution with a mild detergent, as outlined in the previous section. We found that YaxAB derived from these two distinct procedures behaved and looked equivalent (Supplementary Figure 5c and Supplementary Figure 7), opting for this second, more facile route for cryo-EM sample preparation. 2D classification of top-view particles in vitreous ice confirmed the heterogeneous nature of the pores, with C10 particles being the most abundant class (Supplementary Figure 7d).

The cryo-EM structure of the YaxAB complex at 6.1 Å resolution (Fourier Shell Correlation (FSC) = 0.143; Fig. 2b, c and Supplementary Movie 1) reveals the predominant C10 symmetric composition of peripheral and interior rings of YaxA and YaxB, respectively (see Supplementary Figure 8 for details on image processing and map reconstruction). Supplementary Figure 8d highlights the narrow spread of local resolution, ranging from ~5 Å around the core of the head domains, to 6–7 Å in the coiled-coil stalk and foot domains. Despite the medium resolution, the quality and connectivity of the EM map was sufficient to allow accurate assignment of amino acid sequence register in most parts of the model (Supplementary Figure 9). In addition, the protomer arrangement was independently verified by MBP-tag localization and crosslinking followed by mass spectrometry (Supplementary Figure 10). Along its largest dimensions, the C10 symmetric complex spans 160 Å perpendicular and 200 Å parallel to the membrane plane (Fig. 2b). The map accommodates one YaxA–YaxB dimer per radial spoke, in which the head domains form an extended interface, while the coiled-coil stalks remain distinctly apart. Notably, YaxA protomers are not in contact with each other in the pore, but interact instead with two adjacent YaxB subunits in *cis* and *trans* (see below). The amphipol surfactant belt, visible as a disc-shaped density (Fig. 2b, highlighted in yellow), delineates the approximate membrane boundaries. Embedded in the amphipol membrane surrogate, the foot domain helices of YaxA and YaxB protomers form a staggered, 40-meric α-helical annulus (Fig. 2c) with a diameter of 31 Å at its narrowest distance (Fig. 2d). We note that by increasing the desired number of classes for 3D classification, a population of C9 symmetric YaxAB particles could also be identified, which led to a 3D reconstruction at 7.6 Å resolution (FSC = 0.143). In this smaller complex, the subunit arrangement remains largely identical to the predominant C10 pores, revealing some flexibility of the subunits to form assemblies of different stoichiometries (Supplementary Figure 11).

### Pore protomers interact through two interface types

**Fig. 3:**
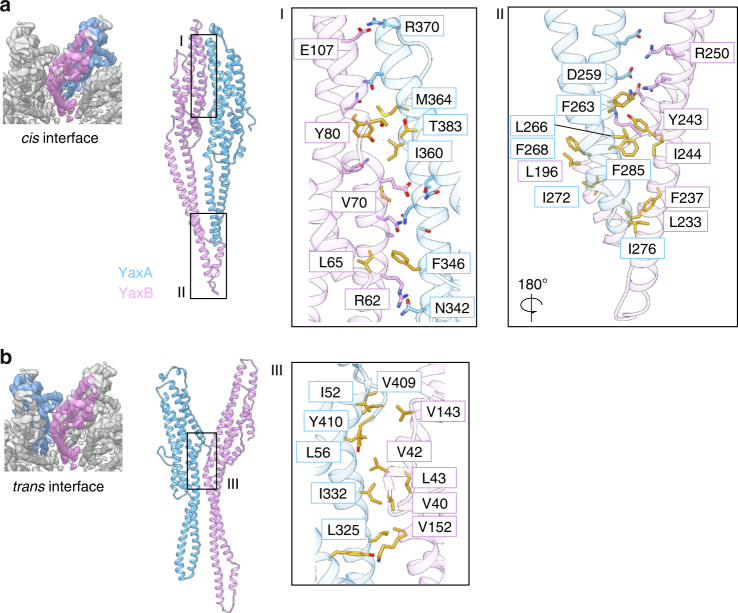
YaxA and YaxB interact via *cis* and *trans* interfaces within the YaxAB complex. **a** Cryo-EM map (left) and modeled structure (right) of the *cis* interface between YaxA (blue) and YaxB (pink) within a radial spoke (*cis*). Boxes labeled I and II indicate contact regions within the protomer pair that are displayed enlarged (right). Residues in interacting proximity are shown as sticks; hydrophobic side chains are colored in gold. Given the medium resolution of the cryo-EM map, the side chains were modeled as ideal rotamers in phenix.real_space_refine. **b** Depiction as in **a**, but of the *trans* interface between YaxA and YaxB from two adjacent radial spokes. Box III indicates the contact region within the protomer pair, colored as in **a**

The largest contact area between pore protomers exists between the head domains of YaxA and YaxB within a spoke (*cis*), burying an average surface of 1476 Å^2^ (Fig. 3a) via a close network of polar and hydrophobic residues. In contrast, no obvious interaction sites connect the coiled-coil stalks. Both proteins resume tight binding at their foot domains, with a series of hydrophobic residues from YaxA (F263, L266, F268, I272, I276, and F285) packing against YaxB (L196, F237, Y243, and I244). These interacting residues in both foot domains are well conserved (Supplementary Figure 12, highlighted in black boxes). Across two spokes (*trans*), YaxA and YaxB interact through a hydrophobic patch at the juncture between head and stalk domains, burying a surface of 1088 Å^2^. This interface (Fig. 3b), which includes the conserved I52/L325, and V40/V42/L43 side chains of YaxA and YaxB, respectively, is likely important for driving complex oligomerization. Furthermore, we observed that the head domains in isolation formed spoked rings, in agreement with the *cis* and *trans* contact sites outlined above (Supplementary Figure 13).

### Characteristics of the YaxAB transmembrane segment

**Fig. 4:**
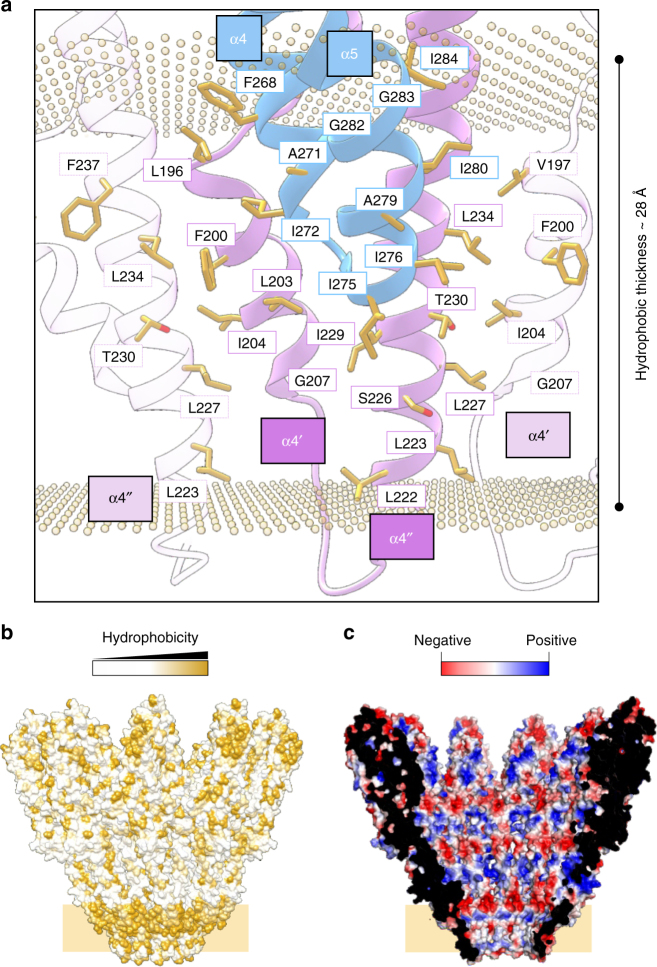
The transmembrane pore is composed of YaxA and YaxB foot domains. **a** Details of a transmembrane segment of the pore. The YaxAB pore model (YaxA: blue, YaxB: pink) was submitted to the PPM server to delineate the buried hydrophobic membrane surface. Shown are foot domains from one *cis*-dimer, as well as the neighboring protomers (transparent ribbons). Membrane exposed residues are colored in gold. The golden spheres demarcate the calculated boundaries of the apolar surface. **b** Hydrophobic surface rendering of the YaxAB model. Gold coloring indicates high hydrophobicity. The golden rectangle delineates the approximate membrane boundaries. **c** Charge distribution of the pore lumen. The surface is rendered by the qualitative electrostatic representation implemented in PyMOL (Schrödinger, LLC)

The putative transmembrane segment of the C10 symmetric YaxAB complex consists of 40 staggered helices, jointly contributed from YaxA and YaxB foot domains (Fig. 2c). Analysis of the pore model using the PPM server^35^ (which positions the protein in a bilayer and calculates the corresponding hydrophobic thickness) confirms the amphipathic nature of the foot domain helices (Fig. 4a). Together, these helices form an apolar surface ~28 Å in length, close to the expected thickness of the membrane interior. Notably, YaxA foot domain helices α4 and α5 cover only about half of this distance, requiring YaxB helices α4′ and α4″ to traverse the entire theoretical bilayer. Consistent with their role as transmembrane helices, the hydrophobic faces of α4′ and α4″ from YaxB are oriented toward the hydrophobic milieu, while their polar faces point toward the pore lumen (Fig. 4b, c). Inside the membrane, two adjacent YaxB protomers form a hydrophobic seal via a conserved α4′ (V197, F200, I204) and α4″ (L227, L234) interface (Fig. 4a). We expect that this characteristic feature might also drive oligomerization between YaxA–YaxB dimers anchored in the membrane.

### Formation of the membrane pore by extrusion of YaxB’s foot

**Fig. 5:**
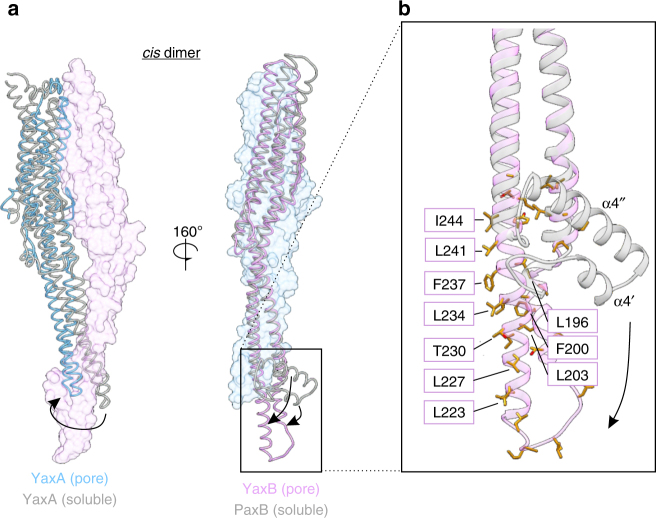
Conformational changes of YaxA and YaxB upon oligomerization. **a** Overview of conformational changes within YaxA and YaxB in context of a *cis* dimer. The interacting protomer is shown in transparent surface representation. Alignment has been carried out between head domains only, given the small rearrangements in this region. Left: monomeric (gray) and protomeric (blue) YaxA. Right: monomeric PaxB (gray) and protomeric YaxB (pink). Arrows emphasize the structural rearrangements during oligomerization. **b** Details of the conformational change in the YaxB/PaxB foot domain transitioning from monomeric to protomeric states. This depiction illustrates the extrusion of the domain’s hydrophobic core. Apolar side chains are colored in gold

Superposition of soluble and protomeric toxin subunits allows for direct visualization of the conformational changes accompanying the transition to a membrane pore (Fig. 5). For YaxA, rearrangements in the head domain remain modest compared with the monomeric protein (Fig. 5a, RMSD of 1.1 Å across 259 Cα pairs). Instead, a twist of the coiled-coil stalk helices α4 and α5 toward the foot domain of the YaxB protomer in *cis* is observed. Because our monomeric YaxB model was incomplete (with the foot domain not resolved in the electron density map), we superposed both monomeric YaxB and PaxB coordinates with protomeric YaxB. This likewise revealed only small conformational changes in the YaxB head domain (Supplementary Figure 2c; RMSD of 1.3 Å across 178 Cα pairs), accompanied by a slight movement of the coiled-coil stalk away from the incoming YaxA. Our monomeric PaxB structure, while complete, might hamper a true comparison with the protomeric YaxB unit, since it is derived from a different species. However, it is noteworthy that the foot domain represents the region of highest sequence conservation between Yersinia and Photorhabdus orthologues (60% sequence identity; Supplementary Figure 12b, highlighted in black box), indeed pointing to a common principle of pore formation. A major rearrangement of the foot domain is apparent from the structural superposition of monomeric PaxB with protomeric YaxB. i) Upon oligomerization, the interface between α4″ and α4/α5 is broken, causing α4″ and its neighboring loops to straighten to one continuous helix with α5. ii) The shorter α4′ helix rotates in the process and likewise incorporates neighboring loops into a longer helix. iii) These rearrangements result in extrusion of the conserved hydrophobic core of YaxB’s foot, whereby α4′ and α4″ become membrane inserted (Fig. 5b). Supplementary Movie 2 illustrates a morph between the two conformations of the YaxB/PaxB foot domain, emphasizing the rearrangement of the amphipathic helices.

### YaxA alone binds to membranes through its foot domain

**Fig. 6:**
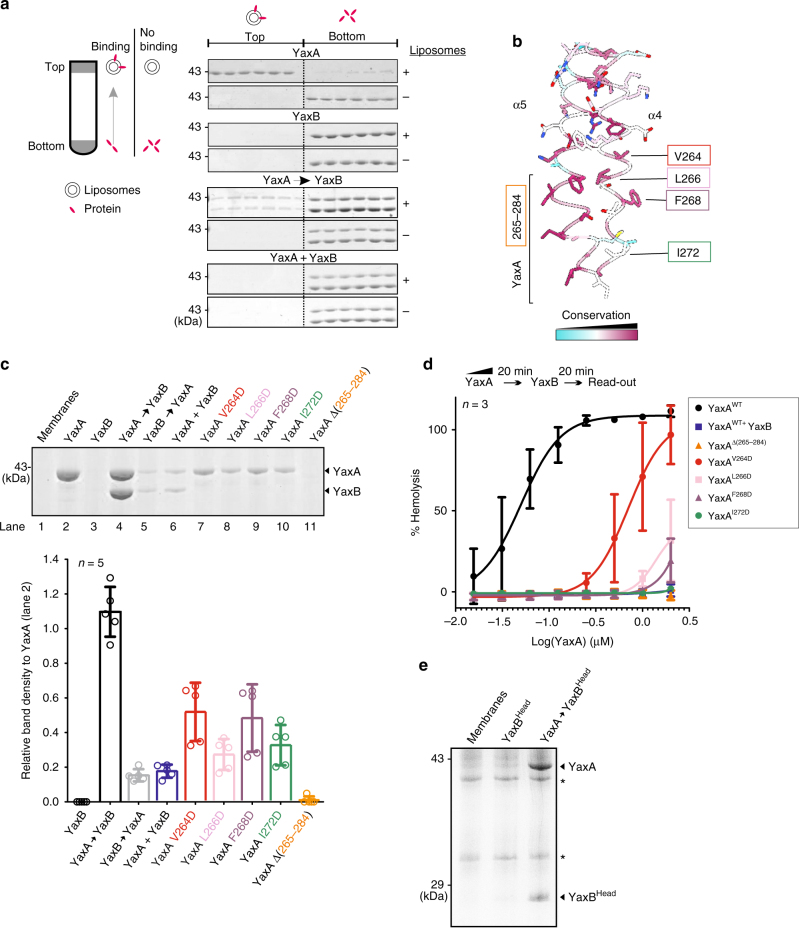
YaxA binds to membranes via its foot domain and recruits YaxB through the head domain. **a** Liposome floatation assay to test membrane-binding abilities of YaxA and YaxB. Left: schematic presentation of the experiment. Right: membrane binding of i) YaxA, ii) YaxB, iii) sequentially added YaxA and YaxB (YaxA → YaxB) and iv) pre-mixed YaxA and YaxB (YaxA + YaxB) assessed by Coomassie stained SDS-PAGE analysis of top and bottom gradient fractions. For each sample, a control run was performed without liposomes. Representative data shown are from one experiment repeated twice. Only a fraction of protein in the YaxA → YaxB sample floated to the top of the gradient in the same time frame, likely reflecting the different behavior of pore-decorated liposomes compared with those bound to YaxA alone. **b** Close-up view of YaxA’s surface-hydrophobic foot domain. Residues targeted for mutation are indicated; a bracket delineates the hydrophobic foot, encompassing residues 265–284. **c** Erythrocyte membrane co-sedimentation assay of WT and mutant YaxA. Top: for each condition, trypsinized erythrocyte ghosts were incubated with the respective toxin component, sedimented, and analyzed by Coomassie stained SDS-PAGE. Bottom: YaxA band intensities relative to the YaxA-only sample (lane 2) were determined densitometrically from the SDS-PAGE scans (Supplementary Figure S15). Data points from five independent experiments (*n* = 5) are shown, along with their means and corresponding standard deviations (SD). **d** Erythrocyte hemolysis assay of WT and mutant YaxA. Erythrocytes were treated first with serial dilutions of WT YaxA or its mutants, followed by YaxB addition. In case of pre-mixed YaxAB, buffer was added for the second incubation step. Hemoglobin release was read at 413 nm and normalized against complete hemolysis by 1% Triton X-100 (100% hemolysis) or a buffer control (0% hemolysis). For each YaxA concentration, the average from three independent experiments (*n* = 3) is presented along with the standard deviation; solid lines correspond to the the fitted dose-response curves. **e** Membrane co-sedimentation assay (conducted as in **c**), demonstrating the ability of membrane-bound YaxA to recruit the isolated YaxB head domain to membranes. Membranes were not trypsinized beforehand; hence protein contaminants (asterisks) are present

The bipartite architecture of the YaxAB complex predicts a functional segregation of YaxA and YaxB in pore formation. Previous reports^27–29^ observed a sequential mode of action for this toxin class in vitro. Using a liposome float assay, we could establish that YaxA binds membranes while YaxB fails to do so (Fig. 6a). In line with previous studies^27, 28^, which used bacterial lysates, YaxAB added sequentially to liposomes bound membranes (which is not the case once YaxAB is premixed).

Looking at a series of conserved and solvent-exposed hydrophobic residues of the YaxA foot domain, we suspected this moiety to be a possible membrane insertion site (Fig. 6b). Thus, we targeted this region for mutagenesis in membrane co-sedimentation assays using erythrocyte ghosts (Fig. 6c; structural integrity of all mutants under study was ascertained by SEC, Supplementary Figure 14). In agreement with the liposome float results, YaxA alone co-sedimented with membranes (Fig. 6c, lane 2), as did a sequentially added sample of YaxA followed by YaxB (Fig. 6c, lane 4). Replacing individual nonpolar residues in the YaxA foot to aspartic acid (V264, L266, F268, and I272, along helix α4) resulted in 2-fold to 3-fold weaker membrane association (Fig. 6c, lanes 7–10). Strikingly, a deletion mutant of most of this domain (Δ265–284; Fig. 6c, lane 11) did not co-sediment with membranes. Reflecting their impaired membrane binding, YaxA foot domain mutants showed reduced hemolytic activity (Fig. 6d), ranging from a 10-fold decrease in potency (V264D) to undetectable activity up to 2 µM (Δ265–284). We attribute the greater loss of lytic activity for the L266D, F268D, and I272D mutants, compared to V264D (despite showing comparably reduced membrane association in Fig. 6c), to these three residues being at the interface of the *cis*-dimer foot domains and thus, likely to be involved in stabilizing the lytic conformation of YaxB (Fig. 3a). In contrast, premixed YaxAB (YaxA^WT^ + YaxB) was lytically inactive up to 2 µM, in agreement with previous reports^27, 28^.

In line with our finding that the isolated YaxA and YaxB head domains form spoked oligomers in solution (Supplementary Figure 13), the YaxB head domain in isolation was sufficient for recruitment to membrane-bound YaxA (Fig. 6e). Together, our biochemical data support a role for YaxA as the initial membrane-interacting subunit of the toxin, able to bring the lytic effector YaxB to the target cell surface.

## Discussion

**Fig. 7:**
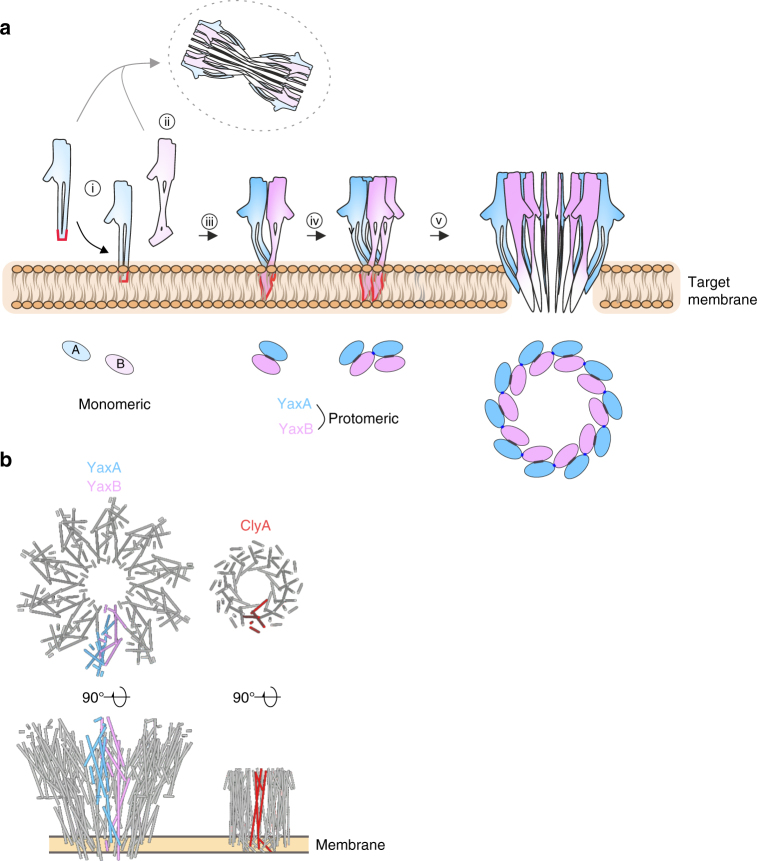
Assembly of the YaxAB pore and comparison with ClyA. **a** Schematic overview of our proposed YaxAB pore assembly pathway at targeted membranes. i) YaxA binds to membranes via its hydrophobic foot, ii) after which YaxB, harboring no membrane binding capacity on its own, is recruited. iii) At this stage, YaxB’s foot domain rearranges to its membrane-inserted state, stabilized by YaxA’s hydrophobic foot. iv) Another membrane-bound YaxAB dimer interacts with the first dimer via the *trans* YaxA–YaxB contact sites. v) Further association of protomers eventually drive formation of the YaxAB pore. The possibility of off-pathway oligomerization, by subunit interaction prior to membrane binding, is indicated by the gray arrow. Red outlines indicate the exposure of membrane-active moieties. **b** Comparison of YaxAB (PDB accession code 6EL1) and ClyA (PDB accession code 2WCD^12^) pore architectures. For a to-scale comparison with the ClyA complex, the structures were tiled in UCSF Chimera. The golden bar indicates approximate membrane boundaries

We have unraveled structures of the soluble components and the pore assembly of the YaxAB cytolysin from *Y. enterocolitica*. The crystal structures show an atypical fold, comprising a head domain reminiscent of ClyA family toxins and pronounced coiled-coil and foot domains unique to the YaxAB system. Together with our cryo-EM model of the YaxAB pore complex (Supplementary Movie 1) and supported by structure-guided biochemistry, we can propose a pathway of pore assembly (Fig. 7a). YaxA, bearing a membrane-binding domain at the tip of its coiled-coil stalk, associates with the target membrane on its own. Membrane-inserted YaxA most likely remains monomeric, since no YaxA–YaxA contacts are found in the YaxAB complex. Uncharacteristic of PFTs, YaxA’s transmembrane moiety is fully solvent exposed in its monomeric state and becomes an integral part of the pore structure upon oligomerization with YaxB. Dimerization with YaxA may induce the opening of the YaxB foot domain (Supplementary Movie 2), whereby amphipathic helices α4′ and α4″ form a tight and highly conserved interface with the YaxA foot helices α4 and α5 inside the membrane. Membrane-bound YaxA–YaxB dimers, as the basic protomeric units, can associate via the exposed contact sites in *trans*. This proposed pathway of pore formation (Fig. 7a) is derived from analysis of the cryo-EM structure and by our biochemical assays discriminating between membrane-binding abilities of YaxA and YaxB.

Past studies have demonstrated that co-expression of XaxAB^27^ (from *X. nematophila*) or YaxAB^28^ produce lytic bacterial lysates, whereas a mixture of subunit containing lysates fails to do so. This latter observation can be explained by the aggregation of YaxAB complexes, when YaxA and YaxB are mixed, which appears to sequester the membrane-active domains of the proteins. While this behavior rationalizes the necessity of a sequential mode of action in vitro (thus avoiding formation of aggregated dead-end pores), it remains open how the toxin would retain lytic activity when the two components are co-expressed in the same cytoplasm. Clarifying this important aspect of YaxAB biochemistry requires future work going beyond our presented structural data.

Previous work on ClyA assembly dynamics^36^ revealed that formation of the dodecameric pore proceeds via association of sterically compatible, membrane-bound multimers (i.e., hexamer with hexamer, pentamer with heptamer, etc.). In light of the structural resemblance of both YaxA and YaxB to pore-protomeric ClyA, we hypothesize that a common pore-forming principle underlies both homo and heteromultimeric PFT systems. In this scenario, multimers of the protomeric membrane-bound YaxA–YaxB dimer would associate in a manner as has been described for ClyA homomers^36^.

Interestingly, the tripartite Nhe and Hbl toxins from *B. cereus*, which belong to the wider ClyA family of PFTs, also display sequential modes of action in vitro, similar to YaxAB^30^. Solving structures of these three-component systems in the future will allow a more comprehensive overview of ClyA-like toxin architectures, a class of PFTs displaying unusual compositional diversity (Fig. 7b).

The precise role of YaxAB in mammalian *Y. enterocolitica* infection still remains elusive. Notably, the toxin was found to be most cytotoxic toward immune cells and influenced the pathology of splenic inflammatory lesions in a murine infection model, pointing to a role in host immunosuppression^28^. The clear functional segregation of the two subunits may be exploited to engineer a dominant-negative YaxA, with a deleted hydrophobic foot domain incapable of engaging target membranes. This latter approach was recently demonstrated on the binary staphylococcal Panton–Valentine leukocidin^37^, and may represent a general strategy to manipulate the activity of multi-component PFTs such as YaxAB.

We anticipate that our findings on the YaxAB system will apply to orthologues from agriculturally relevant insect pathogens like *X. nematophila* (XaxAB) and *P. luminescens* (PaxAB). Promising insecticidal properties of YaxAB orthologues have been confirmed^27, 29, 31^ and future bioengineering efforts could be aided by our structural and mechanistic insights into this class of PFTs.

## Methods

### Recombinant toxin production and purification

The ORFs encoding *Y. enterocolitica* orthologues YaxA and YaxB (YE1984, Gene ID 4715532 and YE1985, Gene ID 4715533, respectively) were amplified from total cDNA of *Y. enterocolitica* serovar 8, biovar 1 (ATCC 23715). The ORF for *P. luminescens* orthologue PaxB (NCBI accession number WP_046395991) was synthesized by Eurofins Genomics. YaxA was cloned as N-terminal His_6_-SUMO fusion into a pRSET-A vector, YaxB and PaxB as N-terminal His_6_ fusions into the same vector containing a TEV cleavage site. All constructs and mutants used in this study were generated by restriction-free cloning, according to the general protocol of Unger et al.^38^. Primers used for cloning wild-type YaxA, YaxB, and PaxB into expression vectors are listed under Supplementary Table 1.

YaxA was expressed in SoluBL21 (DE3) *E. coli* cells (amsbio) grown at 20 °C in 2xTY medium overnight, following induction with 0.5 mM IPTG. YaxB and PaxB were expressed in the BL21 (DE3) strain under the same culture conditions. Selenomethionine substituted protein was produced by the methionine feed-back inhibition method^39^ in M9 minimal medium under otherwise identical culture and expression conditions.

Purification of YaxA, YaxB, and PaxB was carried out according to similar protocols. Cell pellets were resuspended in lysis buffer (50 mM Tris-HCl pH 8.0, 300 mM NaCl, 1 mM PMSF, 1 µg/mL DNase I, 0.2 mg/mL lysozyme) and broken by sonication. Clarified lysate was loaded onto a 5 mL Talon cobalt affinity column (GE Healthcare) equilibrated with buffer A (50 mM Tris-HCl pH 8.0, 300 mM NaCl, 10 mM imidazole) and bound protein eluted in one step with buffer B (50 mM Tris-HCl pH 8.0, 300 mM NaCl, 100 mM imidazole). Pooled fractions were dialyzed overnight at 4 °C against buffer C (20 mM HEPES pH 7.0, 25 mM NaCl) in presence of either SUMO protease (YaxA) or TEV protease (YaxB, PaxB). Next, samples were bound to a 6 mL Resource Q column (GE Healthcare) equilibrated in buffer C and eluted within a linear salt gradient from 25 mM to 1 M NaCl. Peak fractions were further purified on a Superdex 75 16/60 gel filtration column (GE Healthcare) running with buffer D (25 mM HEPES pH 7.0, 150 mM NaCl). Proteins were concentrated to 10–15 mg/mL using 30 kDa MW cut-off centrifugal concentrators (Sartorius) and flash frozen in liquid nitrogen for storage. Purification of selenomethionine derivatized protein followed the same strategy, except for the addition of 2 mM DTT to all buffers. We note that YaxA runs slightly lower on SDS-PAGE gels than its calculated MW of 45.8 kDa—using native mass-spectrometry, we verified that indeed the full-length protein was purified.

### Protein crystallization

Native and selenomethionine substituted YaxA (2 mg/mL) were crystallized using the sitting-drop vapor diffusion method in 0.2 M lithium chloride, 37–40% 2-methyl-2,4-pentanediol, at 4 °C. 0.2 µL of protein solution were mixed with 0.2 µL reservoir solution. Crystals were harvested and plunged directly into liquid nitrogen for storage.

Crystallization of YaxB and PaxB required reductive lysine methylation^40^. Methylated YaxB (14 mg/mL) was crystallized in the hanging-drop vapor diffusion setup at 20 °C. Two microlitres of protein was mixed with 2 µL reservoir solution containing 1.2 M sodium/potassium phosphate. Reservoir solution supplemented with ~4 M sodium malonate^41^ was used as cryoprotectant prior to plunging into liquid nitrogen.

Methylated PaxB yielded sizable crystals only following several rounds of streak seeding in the presence of sodium thiocyanate (NaSCN) as a critical additive. Crystals were grown in hanging-drop setup at 20 °C by mixing 2 µL methylated protein (20 mg/mL) with 2 µL reservoir solution containing 0.2 M magnesium chloride, 0.1 M Bis-Tris pH 5.5, 23–25% PEG 3350, 0.2 M NaSCN. Selenomethionine derivatized crystals were produced by streak seeding native crystal seeds into drops containing derivatized protein. Reservoir solution supplemented with 20% (w/v) 2,5-hexanediol was used as cryoprotectant.

### X-ray data collection and structure determination

Diffraction data were collected on beamline X06SA at the Swiss Light Source (Paul Scherrer Institute, Villigen, Switzerland) using a wavelength of 1 Å for all native crystals. Indexing and data reduction were performed with XDS and XSCALE^42^ (see Table 1 for statistics).

**Table 1:** X-ray data collection and refinement statistics

	YaxA (PDB–6EK7)	PaxB (PDB–6EK4)	YaxB (PDB–6EK8)	YaxA (SeMet)	PaxB (SeMet)
*Data collection*					
Space group	*C*2	*P*2_1_	*P*6_5_22	*C*2	*P*2_1_
Cell dimensions					
* a*, *b*, *c* (Å)	203.8, 24.1, 109.4	104.6, 70.2, 136.9	111.7, 111.7, 169.7	205.1, 24.1, 106.8	105.3, 70.0, 136.3
*α*, *β*, *γ* (°)	90, 113.92, 90	90, 108.41, 90	90, 90, 120	90, 113.97, 90	90, 110.67, 90
Resolution (Å)	50-1.8 (1.9-1.8)	50-2.8 (2.9-2.8)	50-4.0 (4.1-4.0)	30-2.8 (2.9-2.8)	30-3.6 (3.7-3.6)
* R*_meas_ (%)	5.5 (55.0)	6.9 (51.5)	7.7 (95.8)	17.0 (68.1)	15.1 (76.2)
* I* /*σI*	11.5 (2.0)	9.7 (1.8)	13.0 (2.1)	7.9 (2.7)	14.8 (5.0)
Completeness (%)	97.8 (97.6)	95.3 (94.7)	98.7 (99.2)	98.7 (99.2)	99.6 (99.7)
Redundancy	3.7	2.9	5.1	4.8	14.9
*Refinement*					
Resolution (Å)	15-1.8	15-2.8	50-4.0		
No. reflections	43,138	42,429	5351		
* R*_work_/*R*_free_ (%)	19.0/22.8	24.1/26.8	32.1/33.9		
No. atoms					
Protein	3211	10,874	2175		
Ligand/ion	72	4			
Water	240	226			
* B*-factors (Å^2^)					
Protein	55.2	85.9	189.0		
Water, ligand	69.5	83.0			
R.m.s. deviations					
Bond lengths (Å)	0.009	0.004	0.007		
Bond angles (°)	0.9	0.7	1.0		
Ramachandran plot					
Favored (%)	98.6	97.6	98.3		
Allowed (%)	1.4	2.2	1.7		
Disallowed (%)	0	0.2	0		

Each dataset was collected from a single crystal. Values in parentheses are for highest-resolution shell

Experimental phases for YaxA were obtained by SAD methods using a single crystal of selenomethionine derivatized YaxA and a wavelength of 0.979 Å. A total of 500 degrees of data were collected, yielding an anomalous dataset to 2.8 Å resolution. SHELXD^43^ (run within HKL2MAP^44^) identified seven sites and a first electron density map was calculated with SHELXE, in which helical features were clearly visible. Using phenix.find_helices_strands, implemented through the PHENIX GUI^45^, helical densities were fitted with poly-alanine models to ~50% of all residues. Heavy atom sites were polished including the built model in a Phaser MR-SAD^46^ routine. Performing density modification in RESOLVE^47^ and aided by Phaser LLG maps^48^ to visualize selenomethionine positions, the amino acid sequence was gradually traced. Iterative rounds of secondary structure and experimental phase restrained refinement in phenix.refine, with manual rebuilding in Coot^49^, finally yielded a model encompassing ~80% of all residues. At this stage, we incorporated near isomorphous native data to 1.8 Å resolution in model refinement. Translation-liberation-screw (TLS) and individual *B*-factor refinement finally yielded a complete model with converging *R*_work_/*R*_free_ of 19.0%/22.8% and promising bond and angle RMSDs. Validation with MolProbity^50^ confirmed excellent model stereochemistry with no Ramachandran plot outliers.

Phases for PaxB were also obtained by SAD methods using selenomethionine derivatized crystals. Reflections were recorded at a wavelength of 0.978 Å,– to 2.9 Å resolution. Highly redundant anomalous diffraction data allowed the identification of 14 initial selenium sites using PHENIX AutoSol^51^. Subsequent density modification adopting the 4-fold non-crystallographic symmetry (NCS) operators produced a readily interpretable map dominated by alpha-helical features. An initial model of poly-alanine α-helices was produced using phenix.find_helices_strands, automatically placing ~60% of all C-alpha residues of the four-copy asymmetric unit. This model served to refine the selenium sites using Phaser MR-SAD, iteratively improving the map and allowing for sequence tracing during rounds of model rebuilding in Coot. The model was refined with NCS, secondary structure, and experimental phase restraints in phenix.refine. Once the majority of sequence was traced, the model was further refined against the native, near-isomorphous dataset at 2.8 Å. NCS restraints were enabled and finally released once it became clear that residues 188–245 showed marked flexibility within the individual subunits, with density missing in significant portions of this region for one of the four NCS copies. REFMAC 5^52^ was used in the last stages of model refinement, yielding *R*_work_/*R*_free_ values of 24.1%/26.8% and overall good stereochemistry across the four copies in the asymmetric unit, with 0.2% Ramachandran outliers remaining in the model.

The native YaxB dataset was recorded at 4 Å resolution and phased by molecular replacement on the PaxB crystal structure (Chain A). Homology modeling was performed with MODELER^53^ interfaced through UCSF Chimera^54^ and the obtained coordinates applied for Patterson search calculations in Phaser. Though appropriate rotation solutions were achieved, the full-length model was unable to be positioned in its translation, which was due to residues 188–245. Subsequently, these residues were removed from the search model, resulting in a single strong solution, with one copy of YaxB in the asymmetric unit. Following initial rigid-body minimization in phenix.refine, DEN refinement^55^ was implemented to account for re-orientation of the coiled-coil stalk relative to the original PaxB coordinates. Further TLS and grouped *B*-factor refinement in REFMAC resulted in converging *R*_work_/*R*_free_ of 32.1%/33.9% with excellent model stereochemistry. Due to missing density, residues 188–245 remain unmodelled.

### Preparation of detergent-treated YaxAB complex for cryo-EM

Typically, 1 mg each of YaxA and YaxB were combined and incubated at room temperature for 30 min. Cymal-6 was added to 1.5% (w/v) and the mixture incubated at 4 °C for 30 min, after which it was injected onto a Superose 6 column running in buffer E (25 mM HEPES pH 7.0, 150 mM NaCl, 0.05% w/v Cymal-6). Peak fractions were pooled and 3 mg of amphipol A8-35 were (Anatrace) added. The complex was left to incubate for 4 h at 4 °C, after which 20 mg of Bio-Beads (Bio-Rad) were added over night at 4 °C. The next day, amphipol exchanged protein was separated on a Superose 6 column running in detergent free buffer D and used immediately for cryo-EM measurements.

### Cryo-EM sample vitrification and data acquisition

Grids for cryo-EM experiments were prepared using a Vitrobot Mark IV (FEI, Eindhoven). Four microlitres of YaxAB, treated with detergent and amphipol exchanged, at a concentration of 2 mg/mL, was applied onto glow-discharged C-flat 2/1 grids. Prior to vitrification, fluorinated Fos-Choline-8 (Anatrace) was added to the sample in concentrations up to 3 mM to improve the particle orientation distribution. The grids were blotted for up to 4 s, with an offset of −1 to −2 mm at 100% humidity and then plunged into liquid ethane. In total, three datasets were collected on a Titan Krios (FEI) electron microscope operating at an acceleration voltage of 300 kV. Images were acquired with a Falcon-III detector (FEI) in linear mode, at a magnification of 59,000, corresponding to a magnified pixel size of 1.106 Å/pixel. During each exposure, 13 frames were recorded with a total dose of ~60e−/ Å^2^ and a total exposure time of 1 s. 4859 images were automatically acquired using EPU software (FEI), with a defocus of −2 µm. Details of data collection, model refinement, and validation are listed in Table 2.

**Table 2:** Cryo-EM data collection, refinement, and validation statistics

	YaxAB (20-mer) (EMDB-3885) (PDB-6EL1)
*Data collection and processing*	
Magnification	59,000
Voltage (kV)	300
Electron exposure (e–/Å^2^)	60
Defocus range (μm)	1.1–2.5
Pixel size (Å)	1.106
Symmetry imposed	C10
Initial particle images (no.)	178,149
Final particle images (no.)	24,822
Map resolution (Å)	6.1
FSC threshold	0.143
Map resolution range (Å)	4.8–7.0
*Refinement*	
Initial model used (PDB code)	6EK7, 6EK8
Model resolution (Å)	6.1
FSC threshold	0.143
Model resolution range (Å)	4.8–7.0
Map sharpening *B*-factor (Å^2^)	−331
Model composition	
Protein residues	6830
* B*-factors (Å^2^)	
Protein	322
R.m.s. deviations	
Bond lengths (Å)	0.005
Bond angles (°)	0.93
Validation	
MolProbity score	1.87
Clashscore	7.4
Poor rotamers (%)	0.5
Ramachandran plot	
Favored (%)	92.5
Allowed (%)	7.5
Disallowed (%)	0

The Yersinia YaxAB system is a pore-forming toxin of so far unknown structure. Here authors present X-ray and cryo-EM to structures of individual subunits and of the YaxAB pore complex, and find that YaxA binds to membranes first and recruits YaxB for subsequent oligomerization

### Cryo-EM image processing

The image processing workflow was performed with RELION 2.1^56^. Image stacks were motion corrected with the program MotionCor2, after which the contrast transfer function values were estimated and corrected using Kai Zhang’s Gctf. 1009 particles were manually picked to create templates for autopicking. 178,149 particles were automatically selected and sorted through four runs of 2D classification. 113,613 particles were chosen for a run of unsupervised 3D classification, using our negative-stain reconstruction as initial reference. Only the class composed of 10 radial spokes was considered (24,822 particles) and further refined imposing C10 symmetry. After movie refinement and particle polishing, the particles were once again refined. Since the reconstructed map had the wrong handedness, apparent by the unusual left-handed coil–coil density, the refined map was flipped and finally post processed. The final reconstruction used for model building had a resolution of 6.1 Å according to the FSC = 0.143 criterion. Local resolution varied from ~5 Å in the head domains to 6–7 Å in the coiled-coil and foot domains. For validation, the final 3D map was projected in 2D for comparison with the experimental 2D class averages using EMAN2^57^, imposing C10 symmetry and using 5° rotational steps. FSC between the model and the final map was computed using an online tool based on EMAN2 (https://www.ebi.ac.uk/pdbe/emdb/validation/fsc/), which revealed an agreement between map and model up to a resolution of 6.6 Å (FSC = 0.5).

### Modeling the YaxAB pore into the cryo-EM density map

Given the good quality of our cryo-EM map, unambiguous assignment of YaxA and YaxB was straightforward (see Supplementary Movie 1). A full model of YaxB was obtained with the Phyre2 One-to-one threading service^58^ based on the complete model of PaxB (chain A). YaxA and YaxB were first placed by rigid body fitting of the monomers into the density map using Coot. Further implementation of Jiggle Fit and Morphing in Coot positioned the coiled-coils inside the helical densities, highlighting that the foot domain of YaxB needed major remodeling. The connectivity of the map was explicit in almost all regions and bulky side chain densities for tryptophan, phenylalanine, tyrosine, arginine, and lysine were apparent in several instances. This allowed confidence in assigning the amino acid register of our pore model, by implementing our refined high-resolution crystal structures. The model was subjected to several rounds of geometry and *B*-factor refinement in phenix.real_space_refine, including secondary structure and NCS restraints. The final model includes YaxA residues 45–153 and 169–410, and YaxB residues 12–343.

### Liposome floatation assays

Liposomes from bovine heart lipid extract (Avanti Polar Lipids, USA) were prepared by extrusion through 0.1 µm membranes using a mini extruding device (Avanti) in buffer D, following the manufacturer’s instructions. For the liposome floatation assay, 10 µg of protein was added to ~300 µg of liposomes and incubated for 20 min at 37 °C. Addition of the second protein (or buffer D, for single protein samples) was carried out and the sample was incubated for another 20 min at 37 °C. Next, 55% (w/v) sucrose in buffer D was added to the protein/liposome mixture, to a final volume of 800 µL and transferred to open ultracentrifugation tubes (Beckman Coulter, USA). These were carefully overlaid with 2.8 mL 40% sucrose (w/v) in buffer D and finally with 400 µL buffer D. The samples were spun at 200,000×*g* in a Beckman Coulter SW 55 Ti rotor at 4 °C for 4 h. Hundred microlitres were fractioned six times from the top of the gradient, then 2.8 mL removed and six fractions of 100 µL taken from the bottom of the gradient. Fractions were mixed with Laemmli buffer and analyzed by SDS-PAGE and Coomassie staining. Full-size gel scans from one representative experiment are shown in Supplementary Figure 15.

### Erythrocyte membrane co-sedimentation assay

Expired and defibrillated human erythrocytes (purchased from the Blutspendedienst des Bayerischen Roten Kreuzes) were lysed hypotonically in 40 volumes of deionized water and pelleted by centrifugation (5000×*g*, 10 min). The procedure was repeated four times to obtain washed erythrocyte ghosts, which were frozen and stored as 200 mg/mL aliquots. Trypsinization of washed erythrocyte ghosts (to digest erythrocyte membrane protein contaminants) was performed by incubating every 10 mg of ghosts with 20 µL of trypsin solution (in buffer D) at 0.1 mg/mL overnight at 37 °C. A 2-fold molar excess of trypsin inhibitor was added and incubated for 1 h at room temperature, after which the ghosts were pelleted (20,000×*g*) and washed in 1 mL buffer D. The wash was repeated four times. For co-sedimentation assays, ~15 mg of ghosts were incubated with 20 µL of protein (0.3 mg/mL) for 20 min at 37 °C. Twenty microlitres of the second protein (or buffer D for single protein samples) at 0.3 mg/mL was added and further incubated for 20 min at 37 °C. The membranes were pelleted and washed four times in 1 mL buffer D. The final pellet was suspended in 20 µL buffer D, mixed with Laemmli buffer, and analyzed by Coomassie stained SDS-PAGE. Full-size gel scans from each experimental repeat are shown in Supplementary Figure 15.

### Hemolysis assays

Hemoglobin release upon cytolysis was used to measure hemolytic activity of wild type (WT) and mutant toxins. Defibrillated human erythrocytes were washed extensively in PBS and collected by centrifugation (1000×*g*, 5 min). A 2.5% (w/v) suspension was made in PBS and 100 µL dispensed in 96-well format. Erythrocytes were primed with serial dilutions of YaxA (0.0156–2 µM) and incubated at 37 °C for 20 min. Subsequently, 100 µL of YaxB (4 µM) in PBS was added and plates incubated for another 20 min at 37 °C. After centrifugation (300×*g*, 5 min) 20 µL of supernatant was diluted into 180 µL of PBS in a new 96-well plate. Absorption at 413 nm was measured and normalized to PBS and 1% (w/v) Triton X-100 values, for 0% and 100% hemolysis, respectively. Data means from three experiments, performed in four technical replicates each, were plotted using GraphPad Prism software.

### Reconstitution of YaxAB from erythrocyte membranes

Pore reconstitution begun typically by incubating 2 mg of YaxA with 200 mg of erythrocyte ghosts and incubation for 30 min at room temperature with gentle rotation. YaxB was added in 1.5-fold molar excess to YaxA and the mixture was incubated for another 30 min. Membranes were pelleted by centrifugation (5000×*g*, 5 min) and resuspended in 15 mL buffer D. The wash was repeated five times to remove soluble, unbound protein. For detergent extraction of reconstituted pores, YaxAB enriched membranes were resuspended in 1 mL solubilization buffer (25 mM HEPES pH 7.0, 150 mM NaCl, 1.5% w/v Cymal-6) per 100 mg membranes and rotated gently for 30 min at 4 °C. Insoluble material was removed by ultracentrifugation (100,000×*g*, 30 min) and the supernatant concentrated with a 100 kDa MW cut-off centrifugal concentrator. The sample was injected onto a Superose 6 10/30 gel filtration column (GE Healthcare) running with buffer E (25 mM HEPES pH 7.0, 150 mM NaCl, 0.05% w/v Cymal-6). YaxAB eluted in the first peak of the chromatogram shortly after the column void volume (between 9–10 mL elution volume). Fractions from this peak were pooled, concentrated, and 200 µL applied onto a 3.8 mL 10–40% w/v sucrose gradient (25 mM HEPES pH 7.0, 150 mM NaCl, 0.05% w/v Cymal-6, 10–40% w/v sucrose). Centrifugation was performed at 100,000×*g* for 18 h at 4 °C. Two-hundred microlitres fractions were taken from top to bottom of the gradient and those containing YaxAB desalted in buffer E using a 5 mL HiTrap Desalting column (GE Healthcare).

### Negative-stain EM

Membrane-extracted YaxAB pores at 0.1 mg/mL were applied to freshly glow-discharged Formvar-supported carbon-coated Cu400 TEM grids (Science Services, Munich) and stained using a 2% aqueous uranyl formate solution containing 25 mM NaOH (Sample incubation 30–60 s, staining 30 s). Images were acquired at 30,000-fold magnification on a Tecnai Spirit (FEI) operated at 120 kV using either Eagle 4 K (FEI) or TVIPS F416 detectors (Tietz Camera Systems). Automated particle picking and 2D classification were performed using Xmipp 3.0^59^. Based on the 2D averages, 14,600 particles were selected and imported into RELION^60^. Using IMAGIC^61^ the initial model for the negative stain 3D reconstruction was generated from three 2D averages assuming either 10-fold, 11-fold, or 12-fold rotational symmetry. Individual 3D classification runs—without imposing symmetry—using either of the constructed initial models always produced classes with apparent 11-fold symmetry. Thus we can assume that our initial model symmetry did not bias the 3D classification.

### Crosslinking mass spectrometry

Cross-linking experiments of the YaxAB complex, extracted, and purified from erythrocyte membranes, were performed with the amine-reactive MS-cleavable DSBU-linker^62^. A 5–10 µM solution of the purified protein complex was used. The reaction was conducted in 20 mM HEPES pH 7.0, 150 mM NaCl, 0.06% Cymal-6. Freshly prepared stock solution of the DSBU-linker (in DMSO) was added to a final concentration of 7 mM to the protein solution. Cross-linking was conducted for 120 min at 4 °C, before the reaction was quenched by adding ammonium bicarbonate to a concentration of 20 mM. Subsequently, the cross-linked YaxAB complex was digested in solution with trypsin according to the manufacturer’s protocol (Smart Digest, Thermo Fisher Scientific, 70 °C, 30 min). Fractionation of the generated peptide fragments was carried out on an Ultimate 3000 RSLC nano-HPLC system (Thermo Fisher Scientific). Separation of the generated peptide mixtures was performed on an Ultimate 3000 RSLC nano-HPLC system using a 90 min gradient from 0.1% formic acid (FA) to 35% acetonitrile, 0.08% FA. The nano-HPLC system was directly coupled to the Nanospray Flex Ion Source (Thermo Fisher Scientific) of an Orbitrap Fusion Tribrid mass spectrometer (Thermo Fisher Scientific). Data were acquired in data-dependent MS/MS mode with higher energy collision-induced dissociation. Each high-resolution full scan (*m/z* 300–1500, *R* = 120,000) in the orbitrap was followed by high-resolution product ion scans (*R* = 15,000) within 5 s, starting with the most intense signal in the full scan mass spectrum (isolation window 2 Th). To identify cross-linked products, raw data were converted into mgf files using the Proteome Discoverer 2.0 (Thermo Fisher Scientific). Cross-linked products were analyzed with the in-house software MeroX 1.6.0^63^. MS and MS/MS data were automatically analyzed, annotated, and manually verified.

### Analytical ultracentrifugation

Sedimentation velocity experiments were performed in a Beckman XL-I analytical ultracentrifuge using a UV/VIS optical system at a wavelength of 280 nm. YaxAB samples were diluted to a concentration of 0.2–0.5 mg/ml in buffer E, in absence or presence of 0.05% (w/v) Cymal-6. The sedimentation speed was 41,370×*g* (Ti-50 rotor) at a temperature of 20 °C. Data analysis was performed in Sedfit^64^.

### Generation of figures

All protein ribbon diagrams, surface renderings, and cryo-EM density visualization used in figures and movies were made with UCSF Chimera. All figures were prepared with CorelDRAW X8 (Corel Corporation).

### Data availability

Data supporting the findings of this manuscript are available from the corresponding author upon reasonable request.

Atomic coordinates for YaxA (PDB code 6EK7), PaxB (PDB code 6EK4), YaxB (PDB code 6EK8), and the YaxAB complex (PDB code 6EL1) have been deposited in the RCSB Protein Data Bank. The sharpened cryo-EM map of the YaxAB complex has been deposited in the Electron Microscopy Databank under EMD-3885.

## Electronic supplementary material

Supplementary Information

Description of Additional Supplementary Files

Supplementary Movie 1

Supplementary Movie 2
