# Supplementary material for: Structure and mechanism of the two-component α-helical pore-forming toxin YaxAB
Source: Nat Commun. 2018 May 4;9:1806. doi: 10.1038/s41467-018-04139-2 (PMC5935710; doi:10.1038/s41467-018-04139-2)
Supplement: Supplementary file 1 — Supplementary Information [file 41467_2018_4139_MOESM1_ESM.pdf]

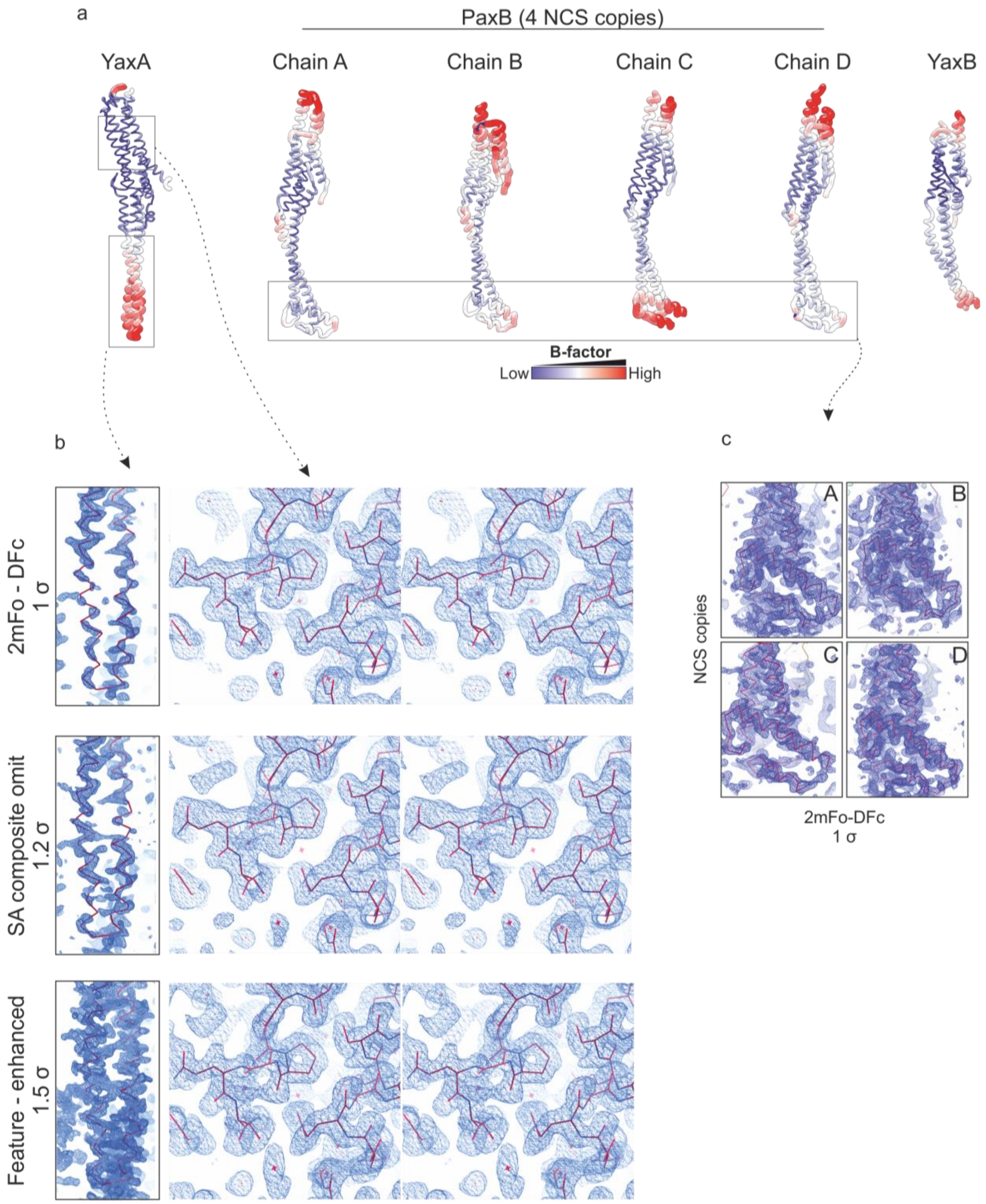

Supplementary Figure 1: Crystal structure B-factor distribution and electron density quality. a) Rendering of YaxA, PaxB and YaxB crystal structures by B-factor values. For PaxB, all four NCS-related copies are shown. b) Quality of the electron density map of two YaxA regions with different average B-factor values: the coiled-coil stalk and foot domains (left) and the head domain (right; stereo pairs are depicted). Shown are three different maps for each region: 2mFo-DFc map (top), simulated-annealing (SA) composite omit 2mFo-DFc map (middle) and feature-enhanced map (bottom). All map calculations were carried out with PHENIX. c) 2mFo-DFc electron density map for the foot domain of the four NCS-related PaxB models.

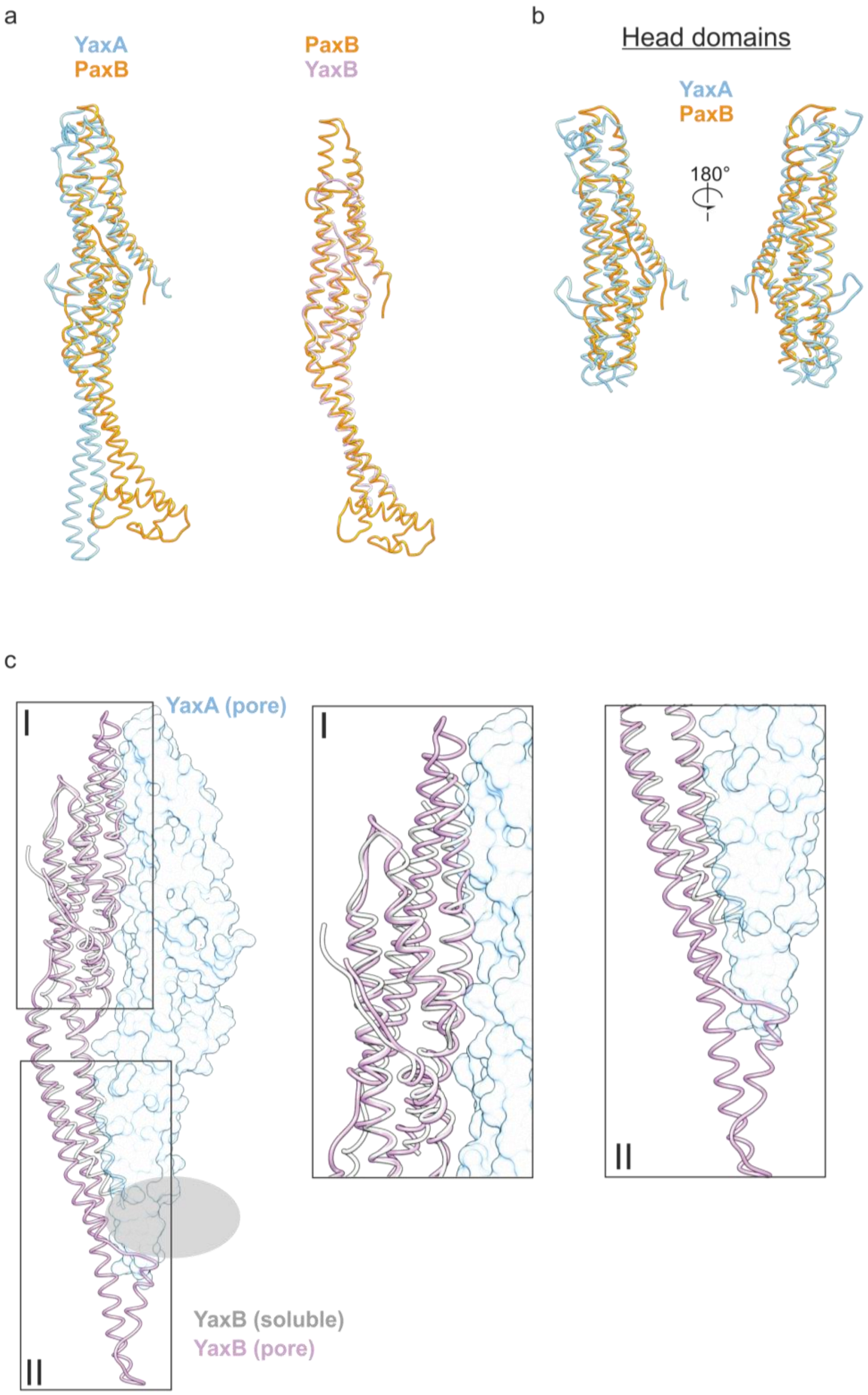

Supplementary Figure 2: Superpositions of YaxA, YaxB and PaxB. a) Structural superposition of monomeric YaxA with PaxB (left) and YaxB with PaxB (right). b) Alignment of the YaxA and PaxB head domains. c) Structural superposition of YaxB (soluble) with YaxB (pore). The interacting YaxA in cis is illustrated as transparent surface (blue). Note the foot domain is not resolved in the monomeric YaxB structure (indicated by a grey oval).

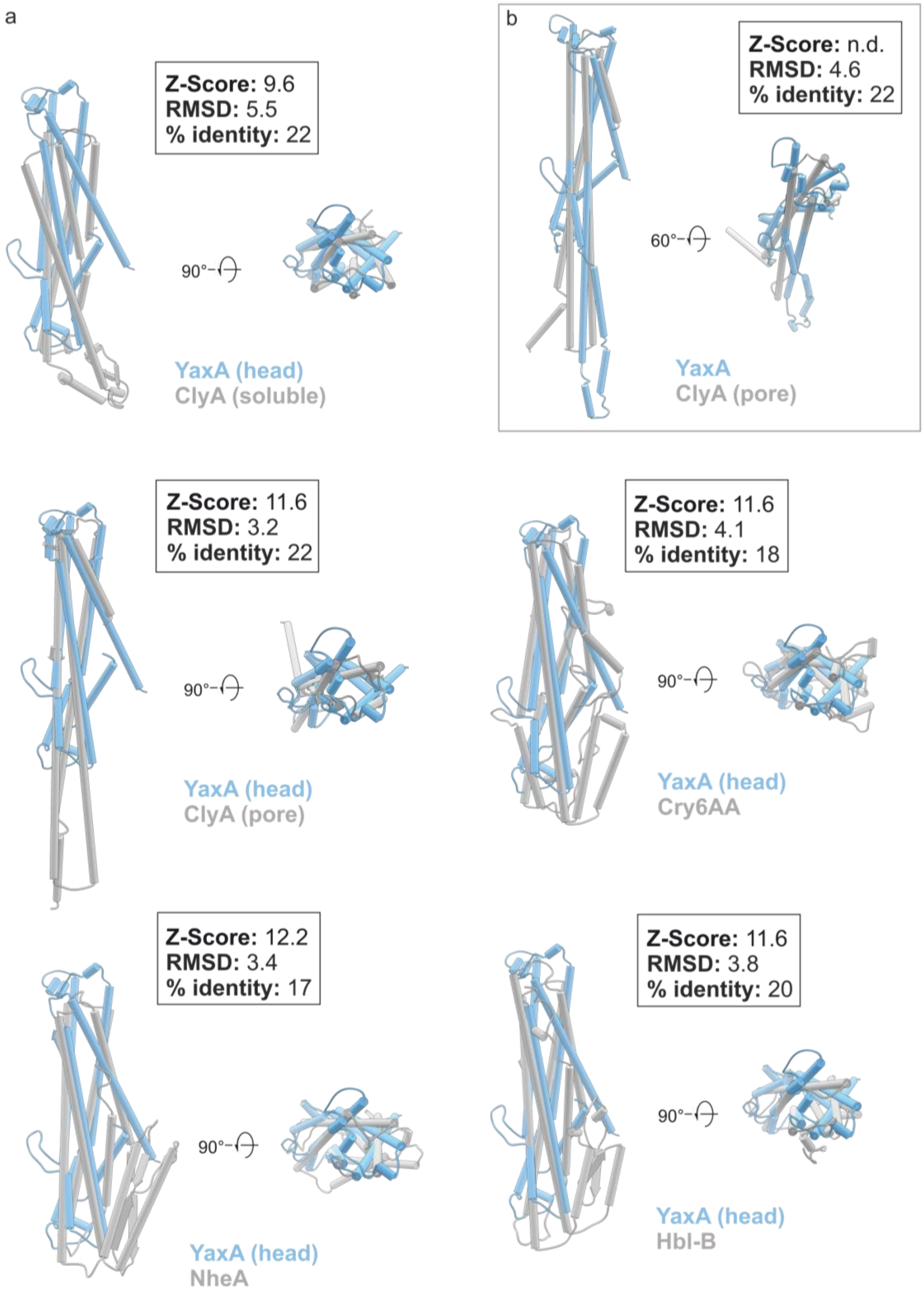

Supplementary Figure 3: Structural superposition of YaxA with ClyA family toxins. a) DALI analysis and alignment of the YaxA head domain (including residues 45 - 237 and 310 - 410) with various ClyA family toxins. PDB codes for aligned structures: 1QOY (soluble ClyA), 2WCD (pore-protomeric ClyA), 4K1P (NheA), 5KUC[1] (Cry6AA), 2NRJ (Hbl-B). DALI Z-score, RMSD (Å) and sequence identity between aligned pairs are presented for each superposition. b) Structural superposition of YaxA (full-length) and pore-protomeric ClyA. The proteins were aligned by their head domains according to a). Notably, pore-protomeric ClyA did not appear as a hit when the full-length YaxA coordinates were used in the DALI search, despite the well-aligning head domains.

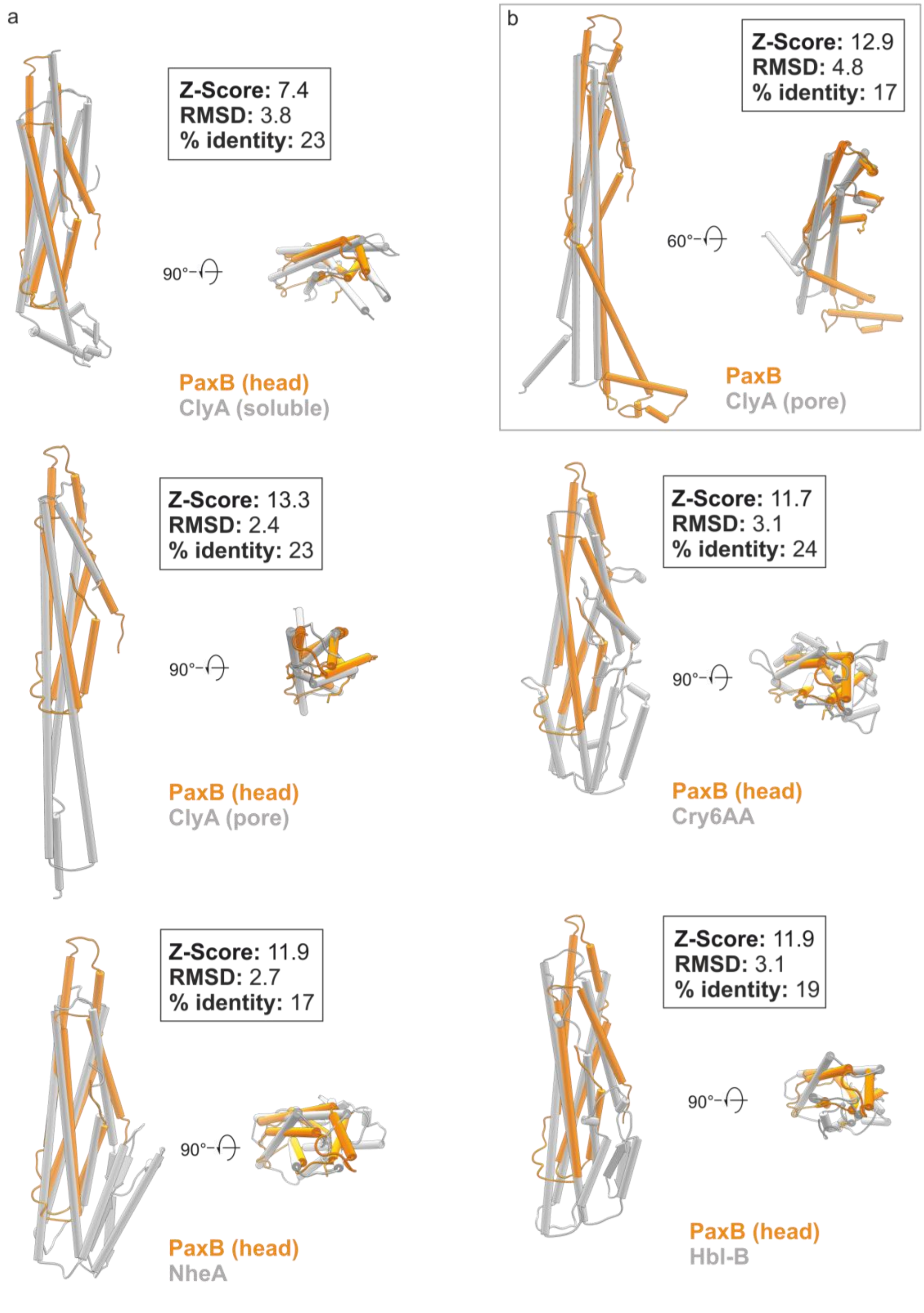

Supplementary Figure 4: Structural superposition of PaxB with ClyA family toxins. a) Alignment of the PaxB head domain (including residues 12 – 153 and 279 – 353) with various ClyA family toxins. b) Superposition of PaxB (full-length) and the pore-protomeric ClyA, as aligned by the DALI server.

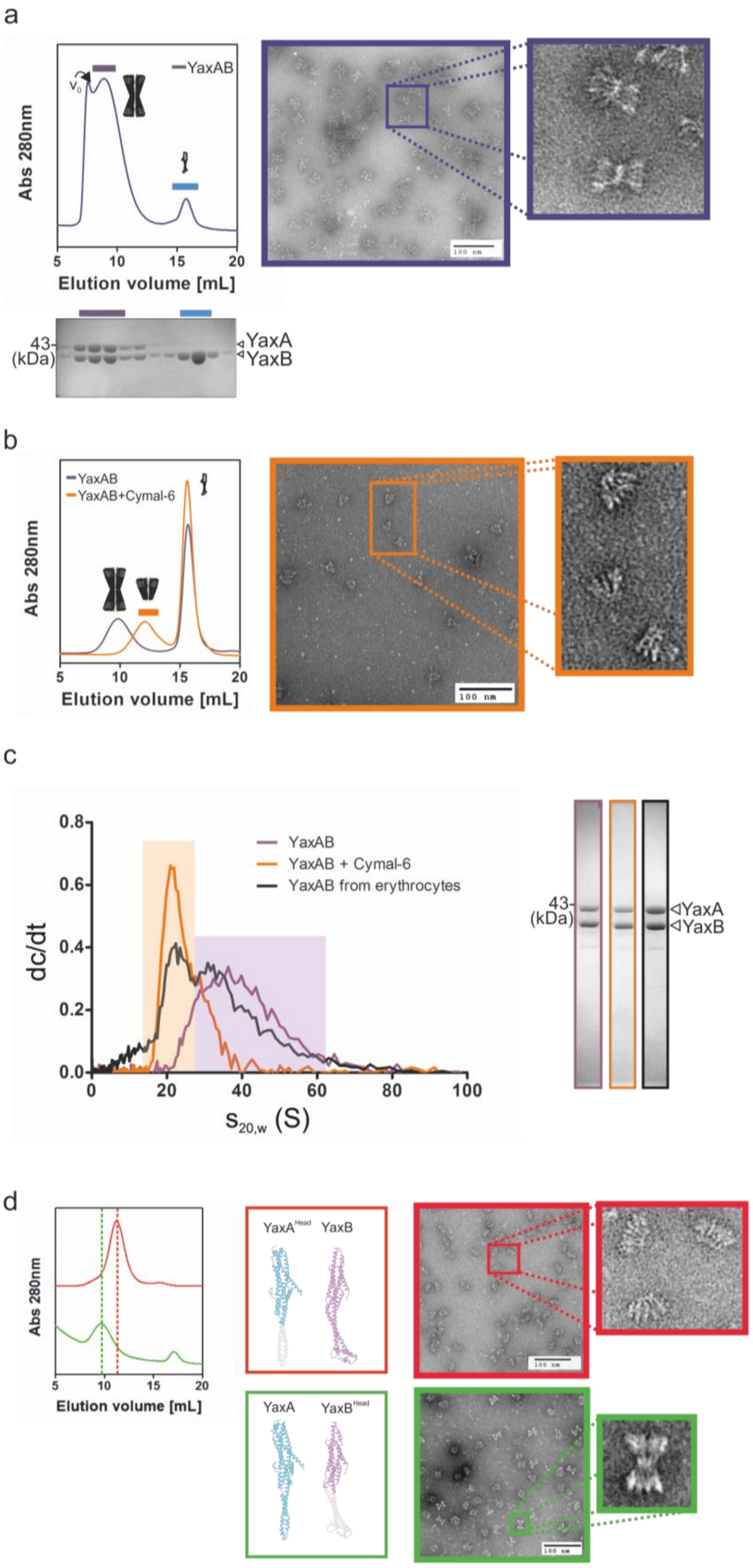
a
YaxAB
V_0
Abs 280nm
5
10
15
20
Elution volume [mL]
100 nm
43
(kDa)
YaxA
YaxB
b
YaxAB
YaxAB+Cymal-6
Abs 280nm
5
10
15
20
Elution volume [mL]
100 nm
c
0.8
0.6
0.4
0.2
0.0
dc/dt
0
20
40
60
80
100
$s_{20,w}$ (S)
YaxAB
YaxAB + Cymal-6
YaxAB from erythrocytes
43
(kDa)
YaxA
YaxB
d
Abs 280nm
5
10
15
20
Elution volume [mL]
YaxA^Head
YaxB
YaxA
YaxB^Head
100 nm
100 nm

Supplementary Figure 5: Detergent dependent aggregation of YaxAB pore complexes is due to YaxA's hydrophobic foot. a) YaxA and YaxB were mixed 1:1 at protein concentrations of 1 mg/mL and 500 µL injected onto a Superose 6 10/300 column. Peak fractions were analyzed by SDS-PAGE and Coomassie staining, depicted below the chromatogram. Fractions from the high-molecular weight peak, containing both YaxA and YaxB, were subsequently imaged by negative-stain TEM. A representative micrograph is shown to the right. b) Comparative gel filtration profiles between detergent-treated (orange trace) and non-treated (purple) YaxAB complexes according to a). For detergent treatment, YaxA and YaxB were incubated (1:1) with 1 % Cymal-6 in buffer D for 20 minutes prior to injection onto the column. Subsequently, gel filtration was run in buffer D supplemented with 0.05 % Cymal-6. Shown on the right is a representative TEM micrograph of the detergent-treated peak fraction. The hourglass-shaped complexes are clearly dissociated compared to a), in accordance with a shift in retention volume as seen for non-treated YaxAB. c) Sedimentation-velocity ultracentrifugation analysis (AUC) of three differently obtained YaxAB complexes (left). In each case, peak fractions from respective gel filtration runs were taken for measurement, verified by SDS-PAGE and Coomassie staining to contain both YaxA and YaxB (right). YaxAB complexes were analyzed from non-treated (purple) and detergent-treated (orange) sample. The complex reconstituted and purified from erythrocyte membranes (black) was also analyzed. As indicated by orange and purple boxes, the complexes partitioned between a ~18-21 S and a diffuse 20-60 S fraction, respectively. d) Aggregation of YaxAB complexes depends on YaxA's hydrophobic foot. Deletion mutants of YaxA (Δ238 - 309) and YaxB (Δ153 - 278), each missing most of the coiled-coil stalk and membrane active foot domains, were combined with the full-length interaction partners and analyzed on SEC as in a). Peak fractions were further imaged by negative-stain TEM.

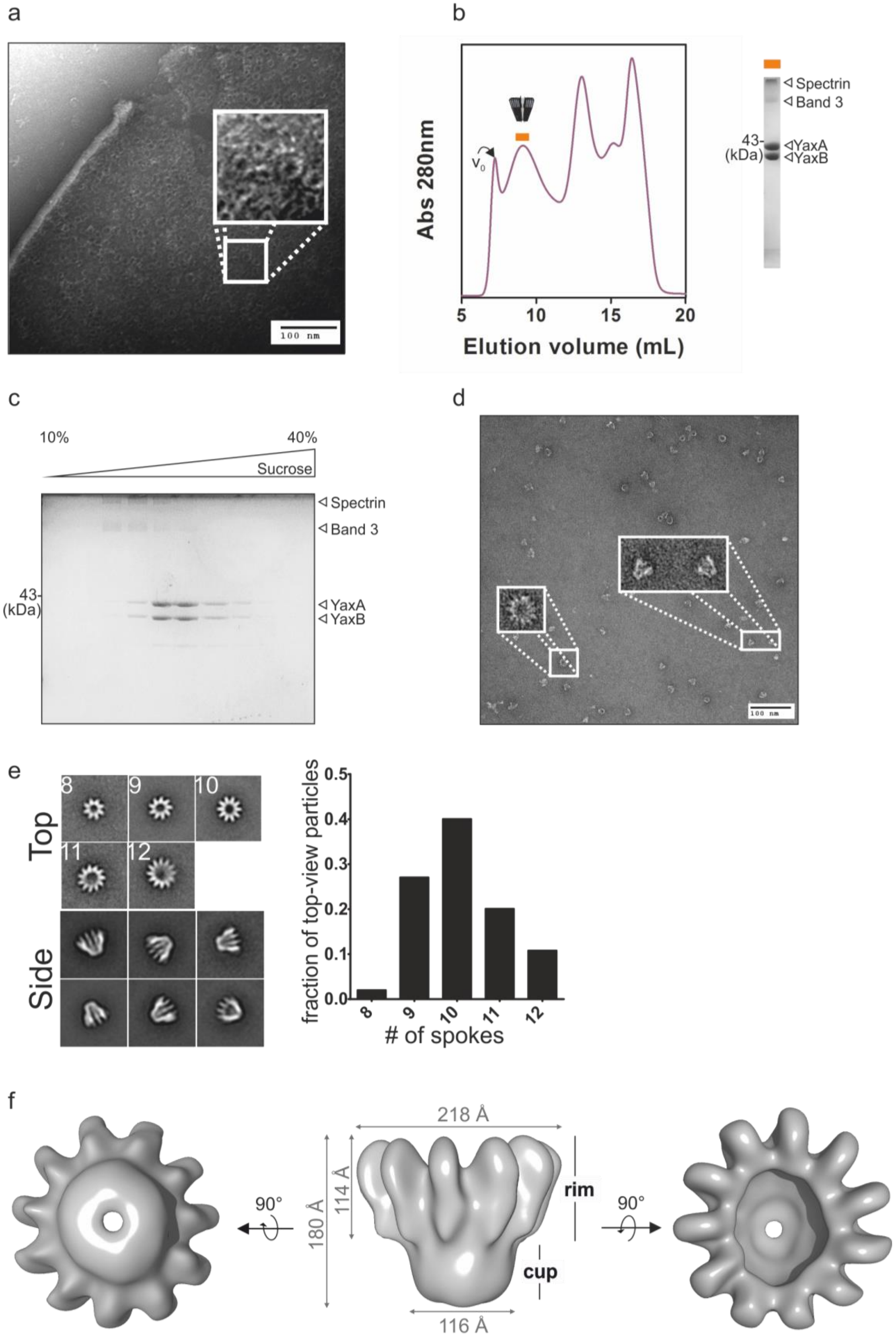

a
100 nm
b
Abs 280nm
V_0
5
10
15
20
Elution volume (mL)
Spectrin
Band 3
43-
(kDa)
YaxA
YaxB
c
10%
40%
Sucrose
Spectrin
Band 3
43-
(kDa)
YaxA
YaxB
d
100 nm
e
Top
Side
8
9
10
11
12
fraction of top-view particles
0.0
0.1
0.2
0.3
0.4
0.5
# of spokes
f
218 Å
114 Å
180 Å
116 Å
rim
cup
90°
90°

Supplementary Figure 6: Negative-stain TEM analysis of membrane-extracted YaxAB. a) YaxA and YaxB were added sequentially onto human erythrocyte ghosts and imaged by negative-stain TEM. The membranes were densely covered in pore complexes (enlarged view). b) YaxAB enriched membranes were solubilized with 1 % Cymal-6 and injected onto a Superose 6 gel filtration column. YaxAB eluted in the first peak, which was verified by SDS-PAGE analysis (shown on the right). c) The Superose 6 fractions were further purified by sucrose gradient centrifugation (10 - 40 % sucrose). Main contaminants spectrin and band 3 anion channel could be separated from the YaxAB complex. d) Representative negative-stain TEM micrograph of the purified YaxAB complex. This sample was also used for XL-MS analysis. e) Gallery of top- and side-view 2D class averages of YaxAB (left) and the distribution of top-view radial spoke numbers (right). On average, top-view particles possessed ten radial spokes. f) Three views from a 3D reconstruction of membrane-extracted YaxAB, bearing apparent C11 symmetry. Symmetry was not imposed during classification and refinement, because some distortion of particle symmetry was expected during negative-staining. This model served as a reference for 3D classification of cryo-EM data.

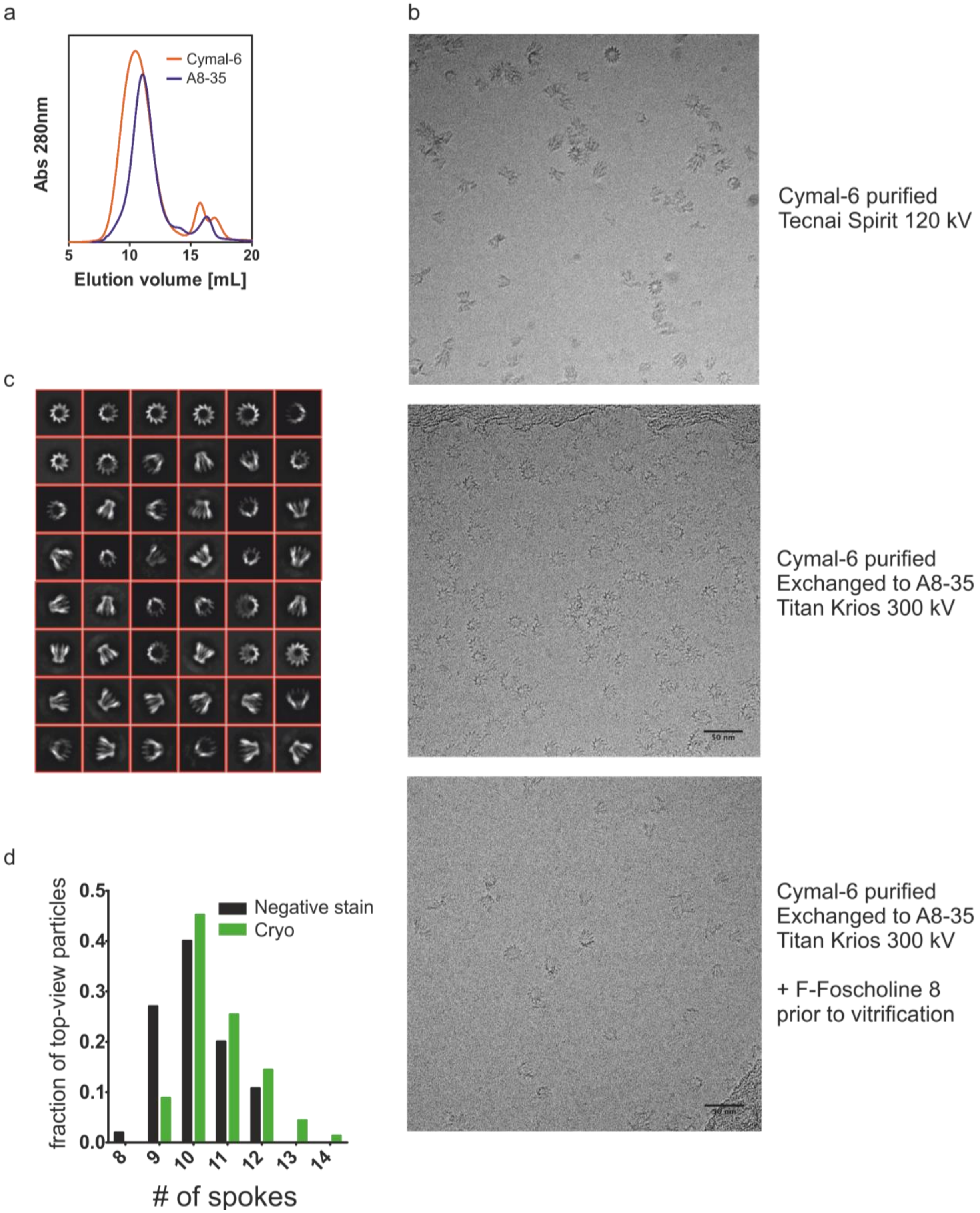

Supplementary Figure 7: Cryo-EM sample preparation and 2D classification. a) Amphiphol exchange of Cymal-6 purified YaxAB as the last step of sample preparation for cryo-EM. Shown are the first SEC trace of detergent-treated YaxAB run in presence of Cymal-6 (orange) and the second SEC run after amphiphol exchange in absence of detergent (purple). b) Representative cryo-EM micrographs of detergent purified (top) and amphiphol exchanged (bottom) YaxAB. In detergent, particles tended to aggregate into long stacks while in amphiphol the complexes remained separate. c) Gallery of 2D class averages after four rounds of classification, starting from 178,000 raw particles selected from automated particle picking. d) Comparison of top-view radial spoke numbers between negative-stain and cryo-EM datasets.

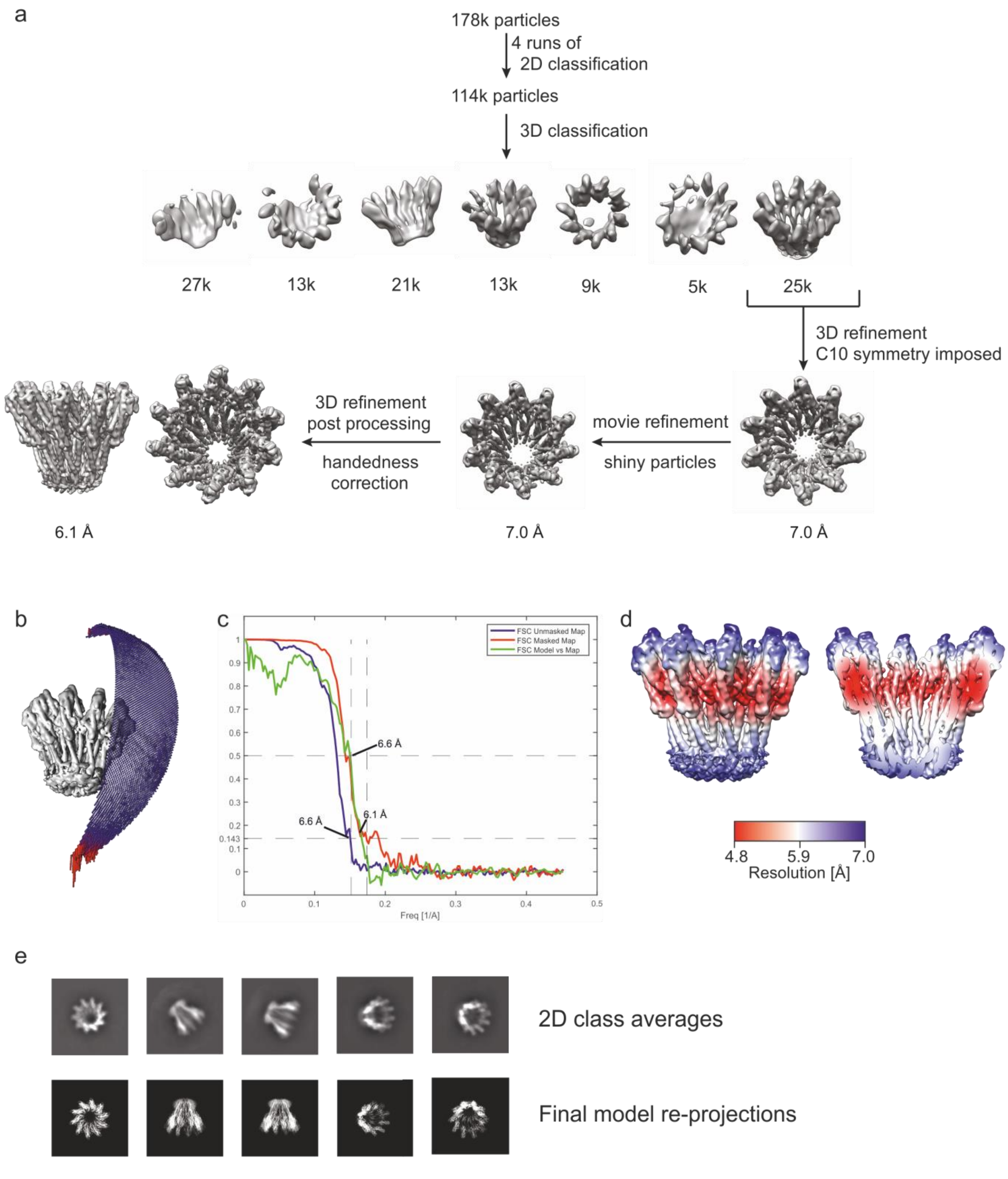

Supplementary Figure 8: Cryo-EM image processing and 3D reconstruction. a) Workflow of cryo-EM data processing and 3D reconstruction in RELION. b) Particle orientation distribution graph for the final reconstruction with C10 symmetry. c) Half-dataset FSC graphs between masked (red) and unmasked (blue) maps are shown together with the model vs. map FSC curve including the final pore model (green). d) Local resolution maps for the final reconstruction. e) Comparison of selected experimental 2D class averages with similar views from 2D projections of the final map.

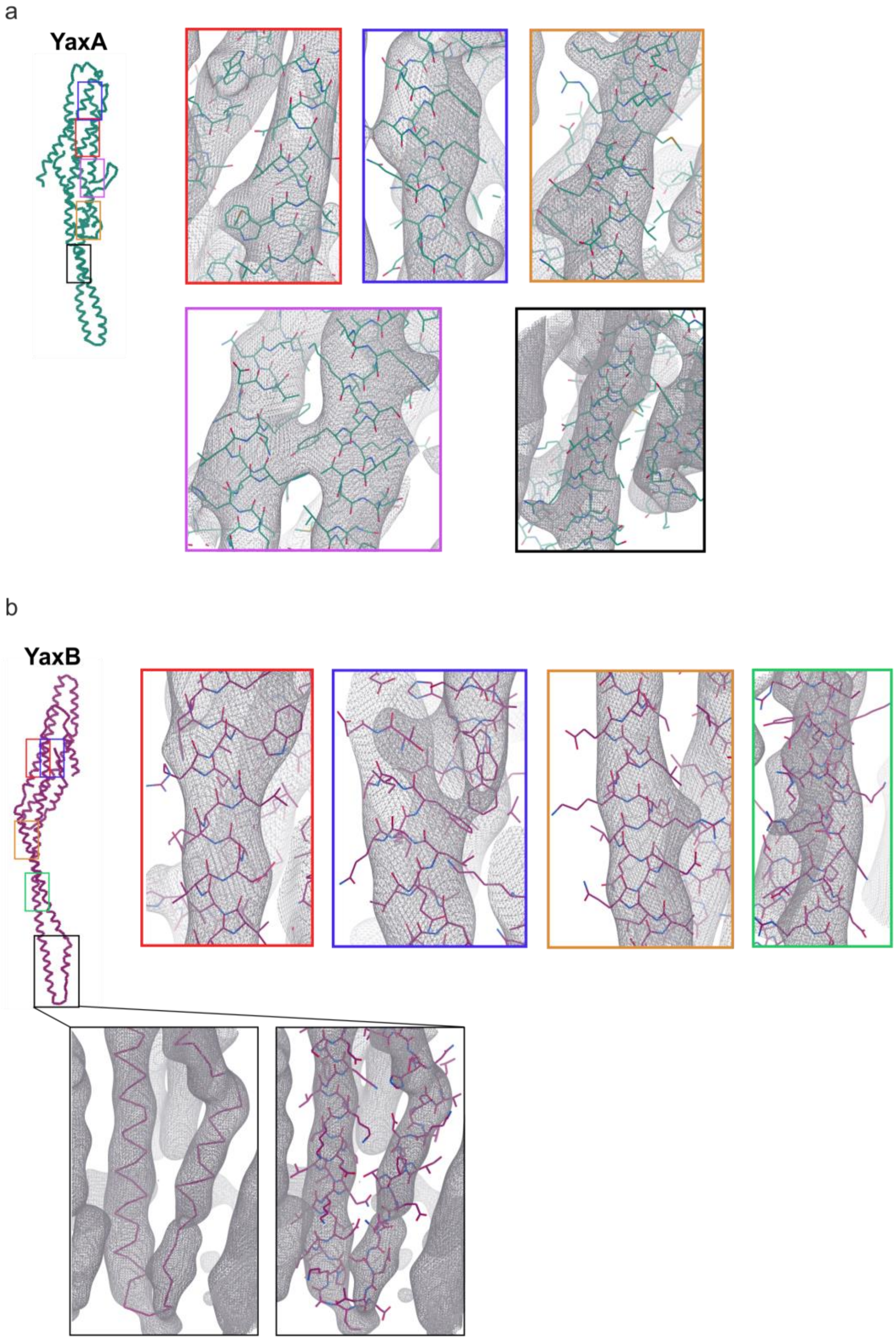

Supplementary Figure 9: Quality of the cryo-EM map and fitted model. Maps are displayed at a contour level of 7σ. a) Examples of the final map at different regions of the protomeric YaxA model. b) Examples of the final map at different regions of the protomeric YaxB model. Density for transmembrane helices α4' and α4'' is overlaid with a Cα-trace (left) and with side-chains modeled (right).

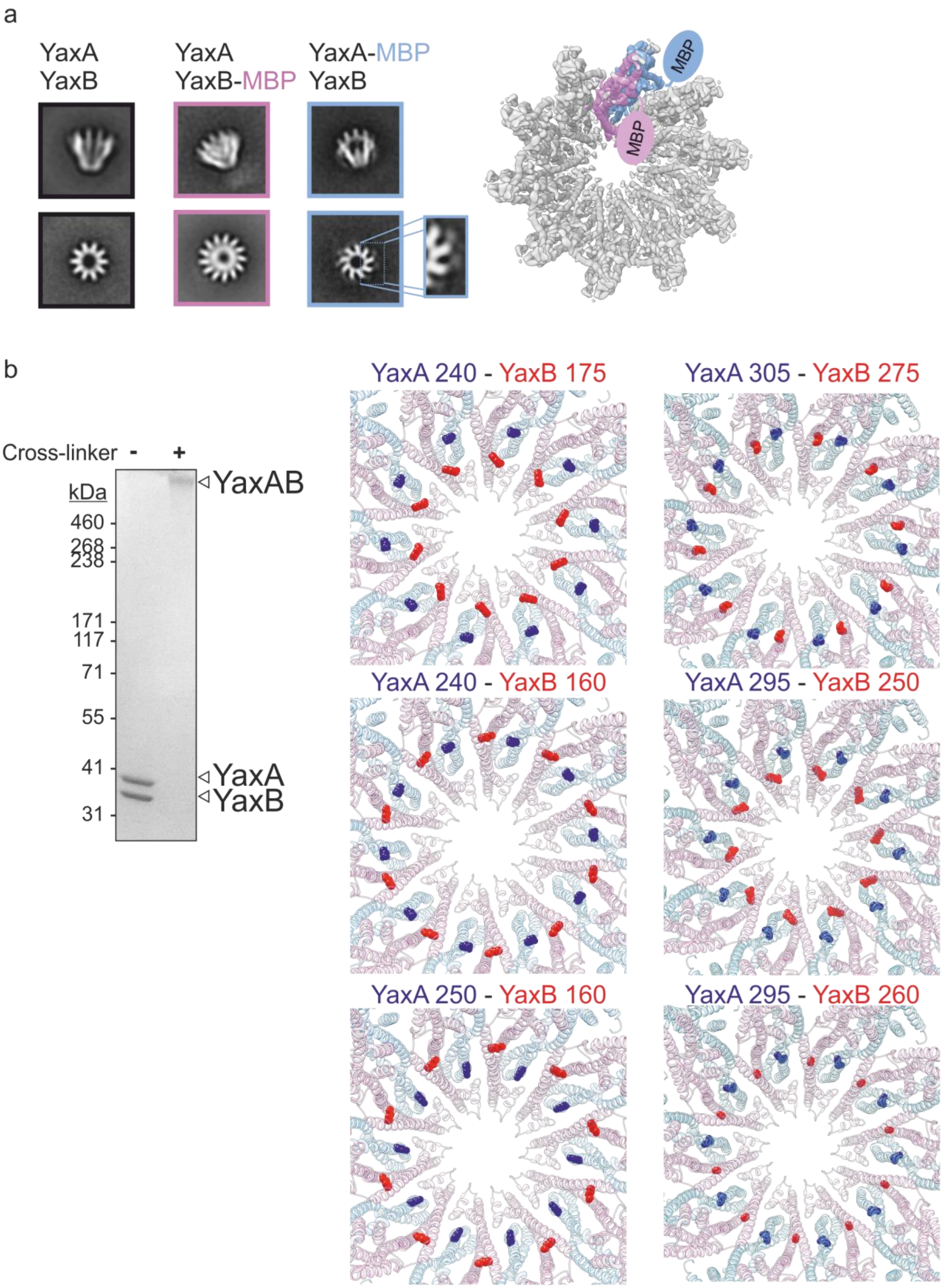
a
YaxA
YaxB
YaxA
YaxB-MBP
YaxA-MBP
YaxB
MBP
MBP
b
Cross-linker - +
kDa
460
268
238
171
117
71
55
41
31
YaxAB
YaxA
YaxB
YaxA 240 - YaxB 175
YaxA 305 - YaxB 275
YaxA 240 - YaxB 160
YaxA 295 - YaxB 250
YaxA 250 - YaxB 160
YaxA 295 - YaxB 260

Supplementary Figure 10: Biochemical validation of the YaxAB model fitted into the cryo-EM density map. a) Localization of the YaxA and YaxB C-termini within the YaxAB complex by MBP-tagging and TEM analysis. C-terminal MBP-fusions of YaxA and YaxB were mixed with the respective un-tagged interaction partner and detergent treated as in Fig. S5b. Negative-stained complexes were imaged by TEM and subjected to 2D classification. Compared with native YaxAB classes (black), both MBP-tagged complexes revealed additional density inside (pink, YaxB-MBP) or outside (blue, YaxA-MBP) the spoked rim. The inset shows an enlarged detail of the additional densities encircling the complex containing YaxA-MBP. Shown on the right is the location of the MBP tags relative to the cryo-EM map of YaxAB. b) XL-MS supports the arrangement of YaxA and YaxB coiled-coils in the YaxAB model. YaxAB (membrane extracted as in Fig. S6) at 5 - 10 μM was crosslinked with the amine-reactive homobifunctional cross-linker DSBU. SDS-PAGE analysis and Coomassie staining (left) confirmed cross-linking efficiency, resulting in one high-molecular weight cross-linked species. A high density of cross-links was identified between the coiled-coil stalks of YaxA and YaxB. Right: Residues of YaxA (red) cross-linked to YaxB (blue) are shown pairwise. The DSBU cross-linker bridges C_α-C_α distances up to 25 Å.

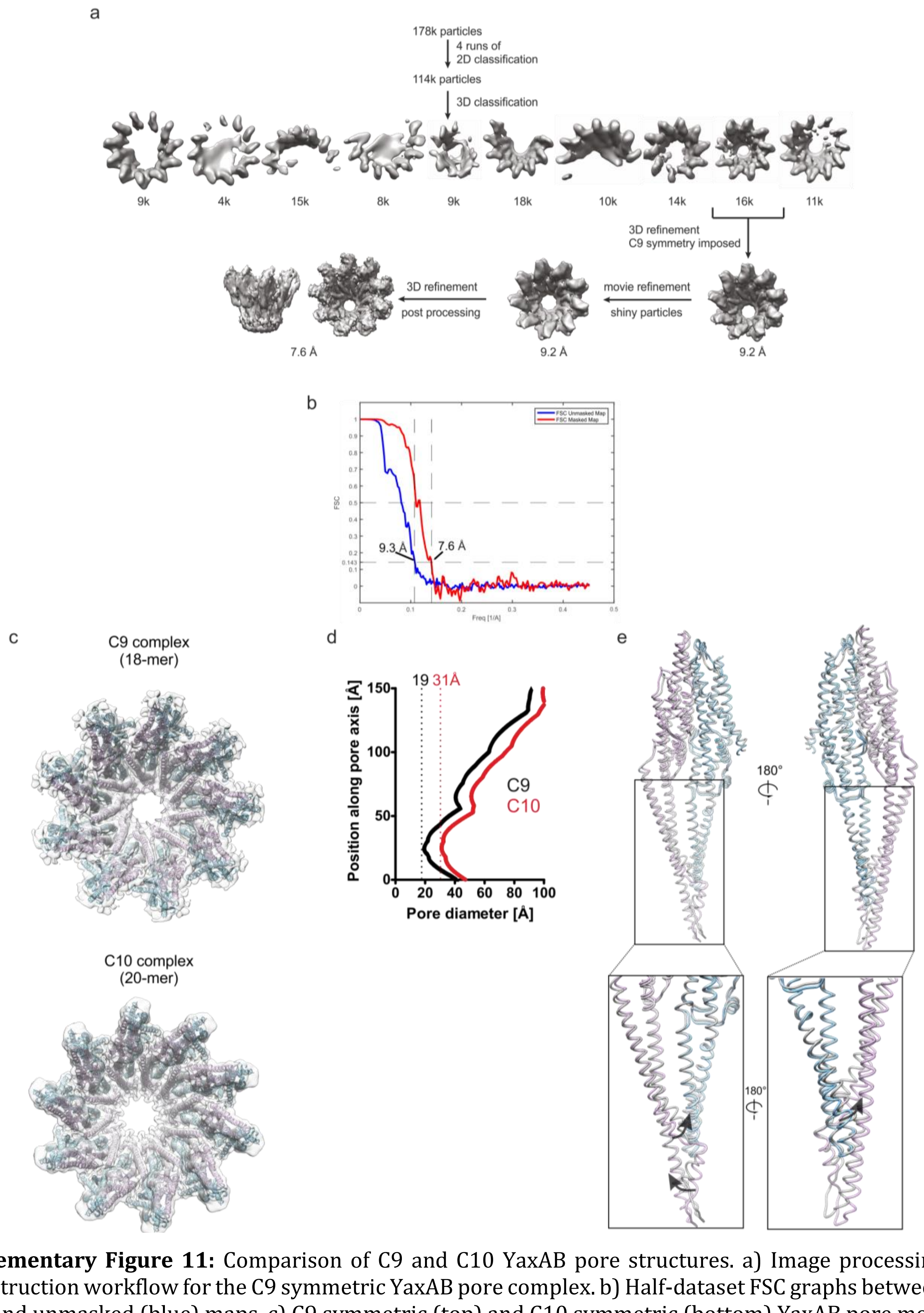

Supplementary Figure 11: Comparison of C9 and C10 YaxAB pore structures. a) Image processing and 3D reconstruction workflow for the C9 symmetric YaxAB pore complex. b) Half-dataset FSC graphs between masked (red) and unmasked (blue) maps. c) C9 symmetric (top) and C10 symmetric (bottom) YaxAB pore models fitted into the respective cryo-EM densities. d) Pore diameter of the C9 (black) and C10 (red) complex plotted against the coordinate along the vertical axis. Calculations have been carried out with the program HOLE. e) Superposition of cis-dimers in the C10 (blue/pink) and C9 (grey) complexes. Arrows emphasize regions of significant rearrangement.

a

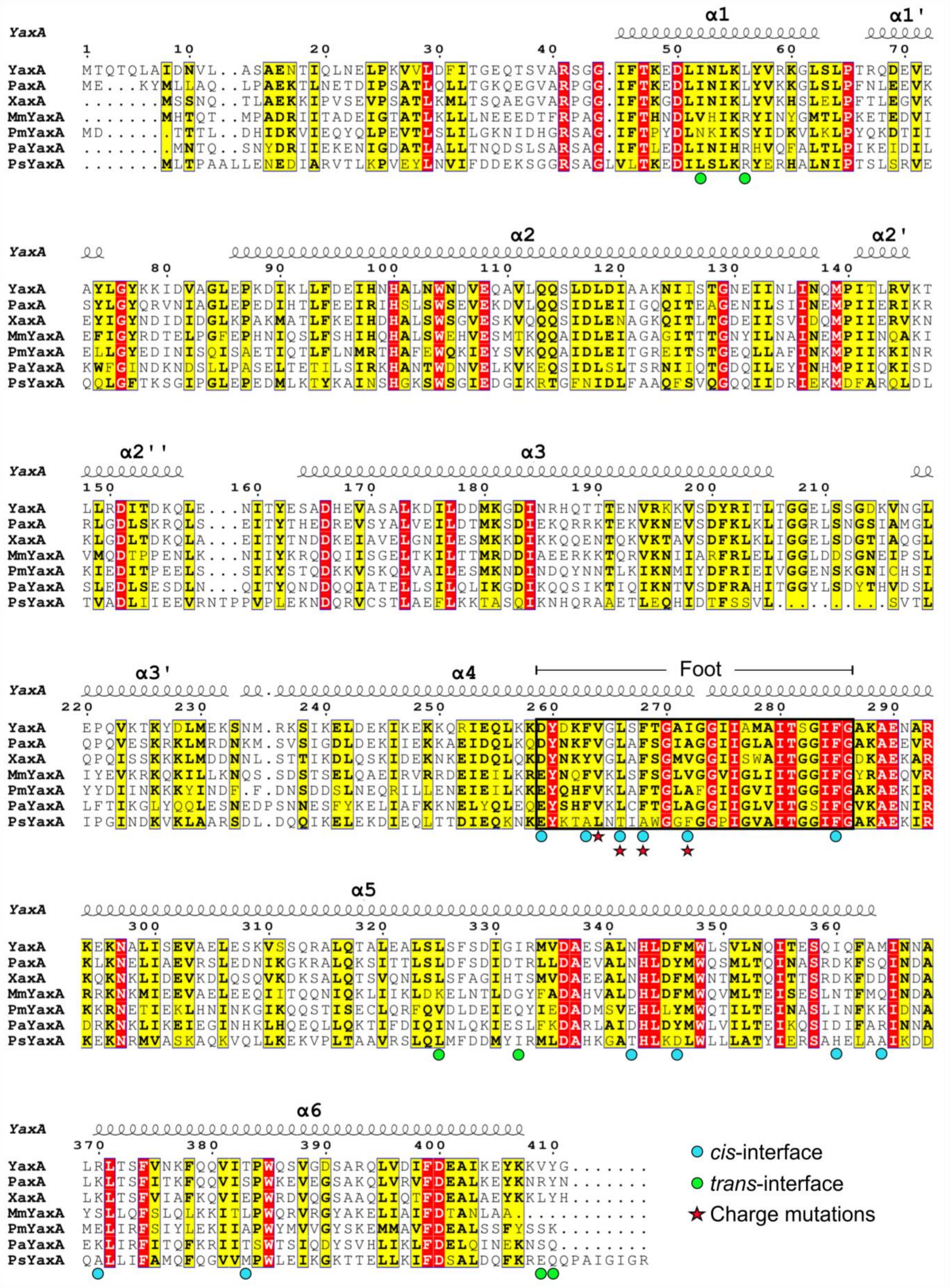
α1
α1'
α2
α2'
α2''
α3
α3'
α4
α5
α6
Foot
YaxA
PaxA
XaxA
MmYaxA
PmYaxA
PaYaxA
PsYaxA
cis-interface
trans-interface
Charge mutations

b

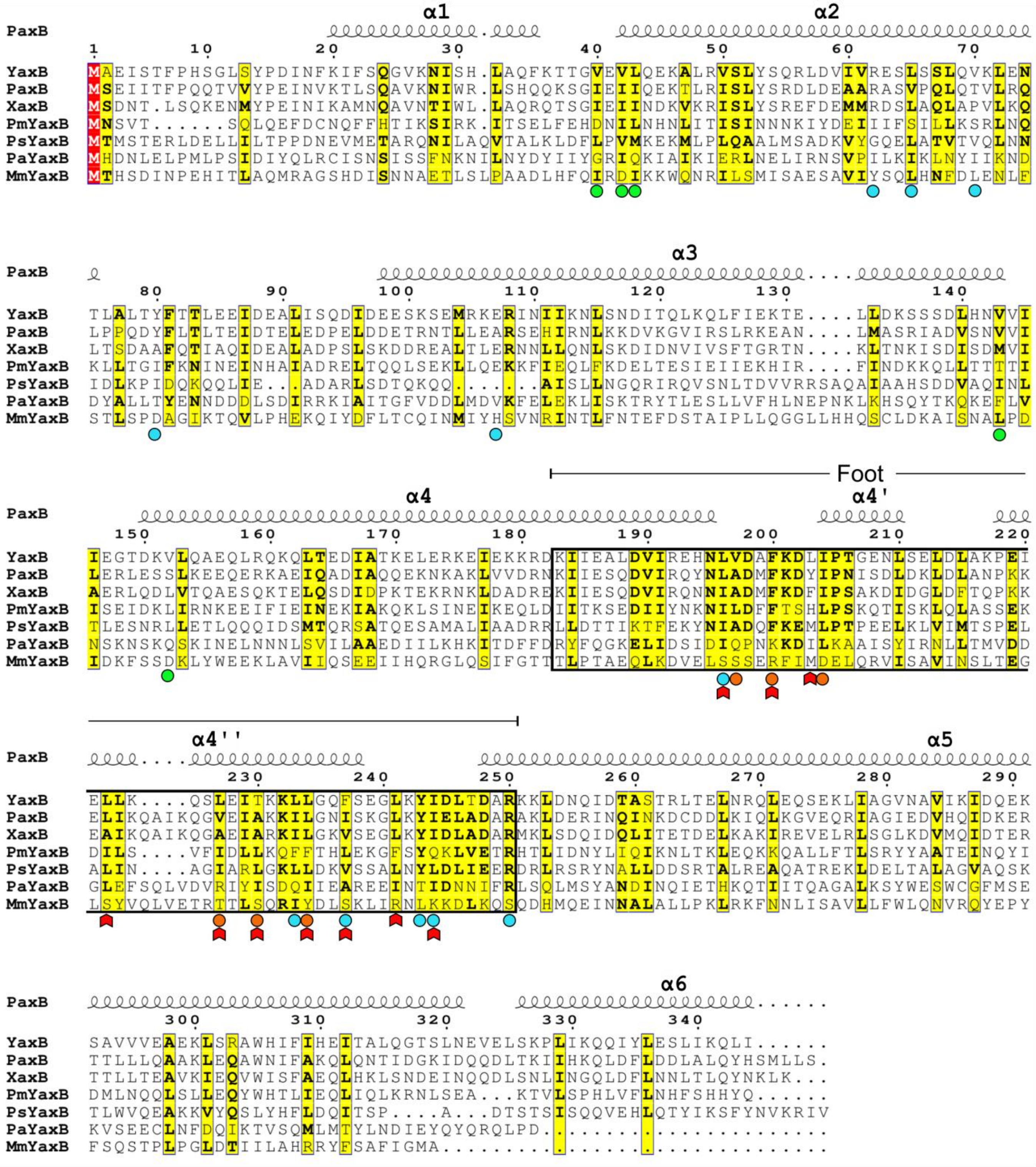

- *cis*-interface
- *trans*-interface
- YaxB-YaxB foot-interface
- Extruded membrane-facing residues

Supplementary Figure 12: Multiple sequence alignment of YaxA and YaxB orthologues. a) Sequence alignment of YaxA orthologues. Sequences correspond to orthologues from Providencia alcalifaciens (PaYaxA), Pseudomonas syringae (PsYaxA), Proteus mirabilis (PmYaxA), Morganella morganii (MmYaxA), Yersinia enterocolitica (YaxA), Photorhabdus luminescens (PaxA), Xenorhabdus nematophila (XaxA). The conserved hydrophobic foot is highlighted by a black frame; positions where charge mutants were introduced are indicated by red stars. Residues engaged in cis-type and trans-type interaction with YaxB are emphasized by cyan and green circles, respectively. Alignments were performed with Clustal Omega[2] and depicted using ESPript[3]. b) Sequence alignment of YaxB orthologues. Sequences were named according to a). The conserved apical foot domain is framed in black. Residues engaged in YaxB-YaxB contacts inside the membrane plane are highlighted by orange circles. Red arrows denote conserved residues facing the lipid milieu as part of the transmembrane segment.

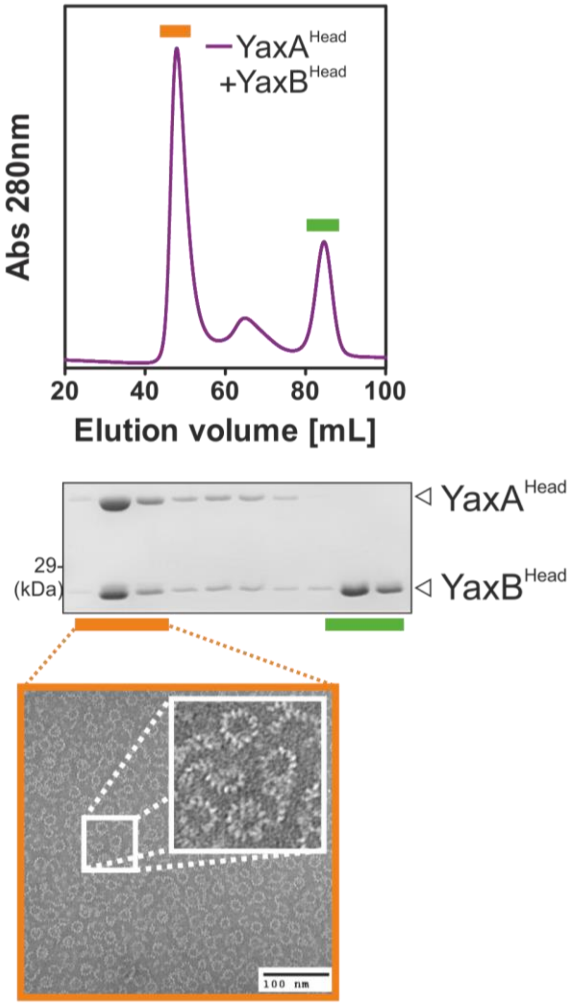

Supplementary Figure 13: The head domains are sufficient for YaxA and YaxB interaction in solution. Gel filtration of a 1:1 (w/w) mixture of YaxA and YaxB head domains revealed their oligomerization in solution (top). Peak fractions were analyzed by SDS-PAGE and Coomassie staining (middle) and fractions containing both proteins were imaged by negative-stain TEM (bottom). The isolated head domains form spoked rings resembling the rims of the full-length YaxAB complex. The inset show enlarged details of raw particles.

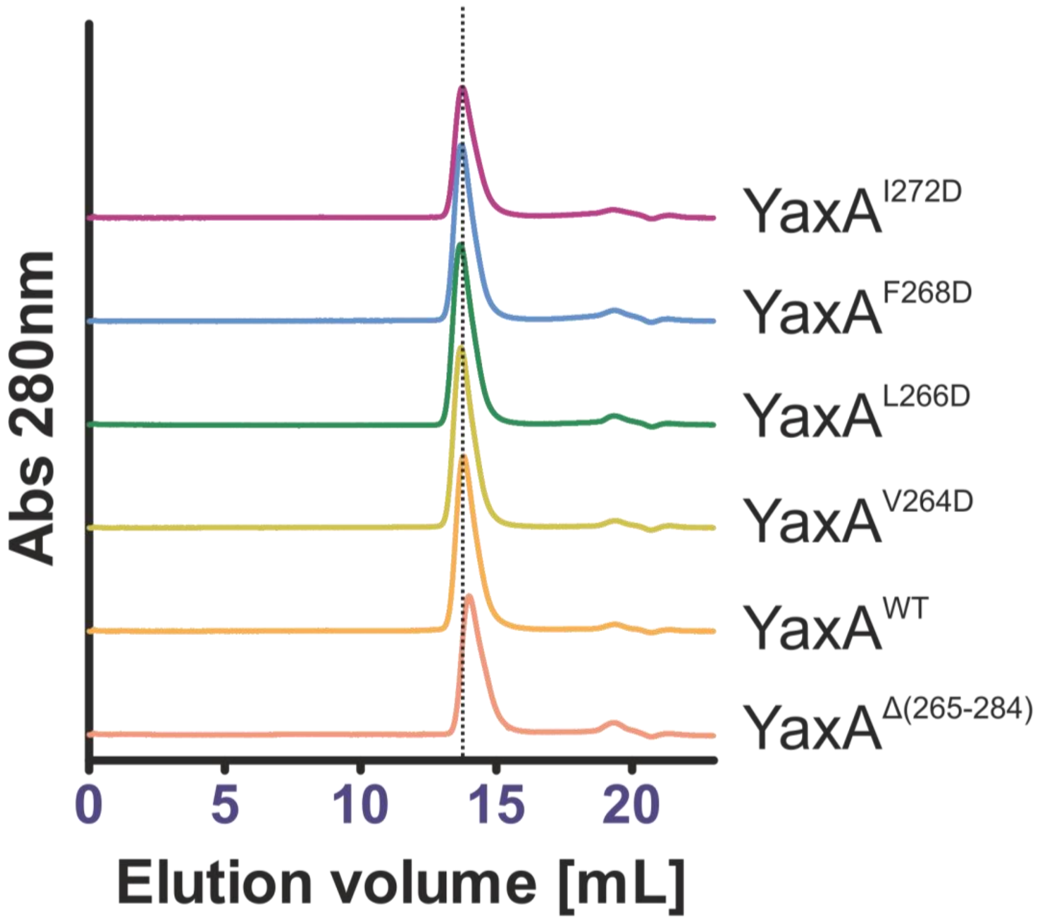

Supplementary Figure 14: Structural integrity of YaxA mutants studied. Gel filtration traces (Superdex 200 10/300 increase) of YaxA mutants used in this work, including wild-type (WT) protein. For each run, 500 µL of protein at ~0.3 mg / mL was injected.

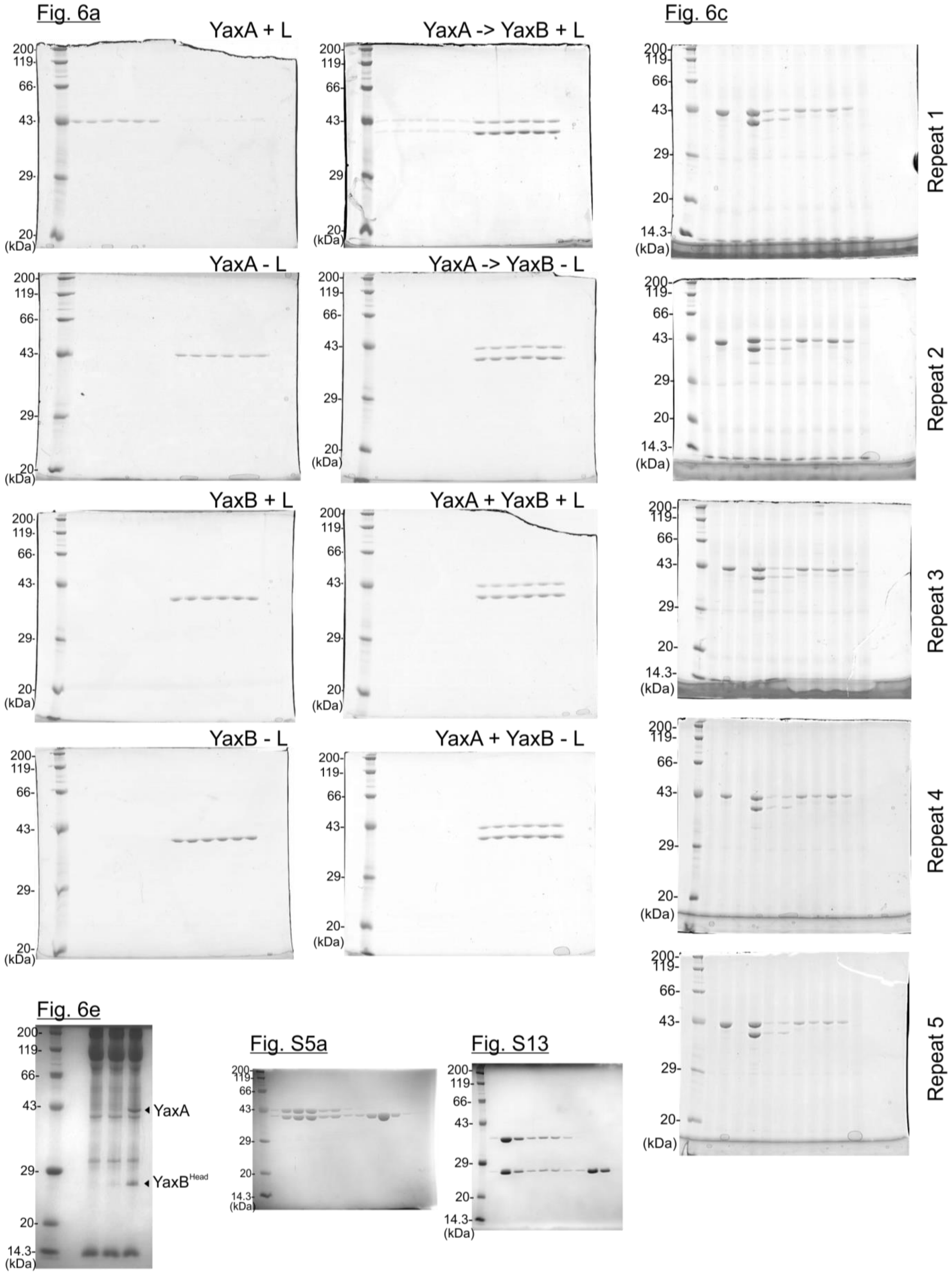

Supplementary Figure 15: Original gel images. Full-size Coomassie stained SDS-PAGE gel scans. The relevant figure, in which portions of the respective gel are shown, is indicated above each scan.

Supplementary Table 1 Primers used for cloning

Underlined: vector complementary; bold: cDNA complementary

Primer name	Primer sequence	Description
pRSET-SUMO-YaxA-F	ATTGAGGCTCACAGAGAACAGATTGGTGGA**GGAATGACACAAACACAATTGGCTATTGATAATGTCTTGG**	Inserting YaxA into pRSET-A-SUMO vector behind His_6-SUMO
pRSET-SUMO-YaxA-R	CTGCAGATCTCGAGCTCGGATCC**TTAGCCATACACTTTTTTGTATTCTTTTATTGCC**	
pRSET-YaxB-F	CCAACGACCGAAAACCTGTATTTTCAG**GGAGCCGAAATAAGCACATTTCC**	Inserting YaxB into pRSET-A vector behind His_6-TEV
pRSET-YaxB-R	CTGCAGATCTCGAGCTCGGATCC**TCAAATCAGCTGTTTGATTAATGACTCTAAATAGATCTGC**	
pRSET-PaxB-F	CCAACGACCGAAAACCTGTATTTTCAG**ATGTCGGAGATTATCACCTTTCCGC**	Inserting PaxB into pRSET-A vector behind His_6-TEV
pRSET-PaxB-R	CTGCAGATCTCGAGCTCGGATCC**CTAGCTCAACAGCATACTGTGATACTGG**	

Supplementary References

1. Dementiev, A. *et al.* The pesticidal Cry6Aa toxin from Bacillus thuringiensis is structurally similar to HlyE-family alpha pore-forming toxins. *BMC Biol.* **14,** (2016).
2. Sievers, F. *et al.* Fast, scalable generation of high-quality protein multiple sequence alignments using Clustal Omega. *Mol. Syst. Biol.* **7,** 539–539 (2014).
3. Robert, X. & Gouet, P. Deciphering key features in protein structures with the new ENDscript server. *Nucleic Acids Res.* **42,** W320–W324 (2014).
