# Supplementary material for: Structure and mechanism of the two-component α-helical pore-forming toxin YaxAB
Source: Nat Commun. 2018 May 4;9:1806. doi: 10.1038/s41467-018-04139-2 (PMC5935710; doi:10.1038/s41467-018-04139-2)
Supplement: Supplementary file 2 — Description of Additional Supplementary Files [file 41467_2018_4139_MOESM2_ESM.pdf]

Description of Additional Supplementary Files

File Name: Supplementary Movie 1
Description: Overview of the YaxAB cryo-EM map and fitted model. The video highlights the cryo-EM map at different contours, whereby the amphipol belt becomes visible. The excellent fit of both YaxA and YaxB is illustrated by the overlay of the cryo-EM map with the fitted models; zoom-ins of head, stalk and foot domains are given. Several parts of the map are shown together with side-chain atoms to illustrate the correct register of the model. The video was created in UCSF Chimera.

File Name: Supplementary Movie 2
Description: Morph between the foot domain conformations of PaxB (soluble) and YaxB (pore). The video illustrates the morph trajectory going from soluble PaxB (YaxB) to pore-protomeric YaxB. The video was prepared using the Morph Conformations utility in UCSF chimera.
